# *Lactiplantibacillus plantarum* P133, a Folate-Producing Probiotic, Ameliorates Cardiac Injury in Hyperhomocysteinemia Mice by Modulating Gut Microbiota and Serum Metabolome

**DOI:** 10.3390/foods15122088

**Published:** 2026-06-09

**Authors:** Wen Dai, Tiantian Jia, Yuanxing Wang, Hengyi Xu

**Affiliations:** 1 State Key Laboratory of Food Science and Resources, Nanchang University, 235 Nanjing East Road, Nanchang 330047, China; 407900230066@email.ncu.edu.cn (W.D.); 407900230114@email.ncu.edu.cn (T.J.); yuanxingwang@ncu.edu.cn (Y.W.); 2 International Institute of Food Innovation Co., Ltd., Nanchang University, Nanchang 330200, China

**Keywords:** hyperhomocysteinemia, *Lactiplantibacillus plantarum* P133, folate, gut microbiota, serum metabolomic, cardioprotective effect

## Abstract

Hyperhomocysteinemia (HHcy) constitutes a significant risk factor for cardiovascular disease. The present study examined the cardioprotective effects and underlying mechanisms of the folate-producing strain *Lactiplantibacillus plantarum* (*L. plantarum*) P133, isolated from traditional fermented pickled vegetables, in a murine model of HHcy induced by a methionine-choline-deficient diet. The findings revealed that administration of P133 significantly reduced serum homocysteine concentrations and improved cardiac function, as evidenced by decreased serum cardiac enzymes (AST, LDH, Ctnt, Ctni), mitigated myocardial histopathological damage, and lowered oxidative stress levels (e.g., decreased MDA). Mechanistically, P133 appears to provide dual protective effects: firstly, it functions as an intrinsic source of folate, thereby mitigating disturbances in one-carbon metabolism; secondly, it influences the composition of the gut microbiota, significantly enhancing the prevalence of beneficial taxa such as Muribaculaceae, and modifies the serum metabolomic profile by increasing favorable metabolites like indoleacetic acid, which correlate strongly with attenuated cardiac injury. These synergistic effects are associated with attenuated cardiac injury. Therefore, *L. plantarum* P133 emerges as a promising probiotic candidate for the prevention and treatment of cardiac damage related to HHcy via a multifaceted intervention approach.

## 1. Introduction

Hyperhomocysteinemia (HHcy) is a prevalent metabolic disorder defined by serum homocysteine (Hcy) levels equal to or exceeding 15 μmol/L [1]. A meta-analysis encompassing approximately 340,000 individuals estimated the prevalence of HHcy in China at 37.2% [2,3]. Furthermore, the data indicated a progressive increase in prevalence over successive years, highlighting this condition as a significant public health concern. The pathogenesis of HHcy is multifactorial, involving dietary influences, genetic predispositions, nutritional status, and the presence of comorbid conditions or pharmacological agents [4]. In the absence of timely intervention, sustained elevated Hcy levels can precipitate a range of chronic complications, including cardiovascular diseases and chronic kidney disease, thereby posing substantial risks to public health [5,6]. Current therapeutic strategies predominantly emphasize lifestyle modifications, including dietary regulation and consistent physical activity, complemented by pharmacological supplementation with vitamin B9 and vitamin B12 [7]. Nevertheless, conventional monotherapeutic approaches have limitations in effectively managing the complex metabolic disturbances associated with HHcy. Therefore, the development of safer and more efficacious treatment modalities for HHcy and its sequelae remains a critical research priority.

Dietary factors play a pivotal role in the development of HHcy [8]. The methionine-choline-deficient (MCD) diet model induces HHcy rapidly and consistently by concurrently restricting methionine and choline, two nutrients essential for homocysteine metabolism. This model not only faithfully replicates the HHcy condition but also frequently induces hepatic steatosis, thereby closely reflecting the clinical manifestations of metabolic syndrome, which often coexists with cardiovascular pathology, underscoring its clinical relevance [9,10,11]. Empirical evidence suggests that elevated homocysteine functions as an independent risk factor for cardiovascular disease by exacerbating cardiac injury through mechanisms including oxidative stress, inflammation, and endothelial dysfunction [12,13]. Traditional single-modality interventions are insufficient to comprehensively mitigate these multifaceted pathogenic processes.

In recent years, the relationship between gut microbiota and metabolic diseases has attracted considerable scholarly interest. Emerging findings indicate that commensal gut microbiota may modulate HHcy and its associated complications via multiple mechanisms. Notably, propionate and butyrate generated through microbial metabolism exert protective effects by activating GPR43 and GPR41, suppressing the NF-κB inflammatory pathway, and enhancing the integrity of the intestinal barrier [14,15]. Liu et al. demonstrated that dietary imbalances, such as high-methionine or MCD diets, significantly alter gut microbiota composition and further aggravate metabolic dysfunction and organ damage [16]. Given the limitations inherent in conventional treatments and accumulating evidence linking gut microbiota dysbiosis to the pathogenesis of diet-induced HHcy, we propose that therapeutic strategies aimed at restoring gut microbial homeostasis, in conjunction with folate supplementation, may constitute a novel and promising approach for preventing and mitigating HHcy-related cardiac injury.

Empirical studies suggest that certain strains of *Lactiplantibacillus plantarum* (*L. plantarum*) can suppress myocardial inflammation by producing bioactive compounds, including indole metabolites and short-chain fatty acids [17]. Additional research has reported that *L. plantarum* alleviates metabolic cardiac damage in murine models through modulation of the gut microbiota, exertion of anti-inflammatory and antioxidant effects, and enhancement of intestinal barrier integrity [18,19]. Moreover, specific strains of *L. plantarum* are capable of synthesizing folate, a critical coenzyme involved in Hcy metabolism [20,21]. Folate facilitates the remethylation of homocysteine to methionine, thereby directly participating in the regulation of homocysteine levels [22]. Consequently, folate-producing lactic acid bacteria may provide synergistic cardioprotective effects in the context of HHcy-induced cardiac injury. However, the efficacy and mechanistic basis of these protective effects have not been comprehensively characterized.

This study aims to investigate the cardioprotective effects of the folate-producing *L. plantarum* strain P133 against HHcy-induced cardiac injury in a murine model, as well as to elucidate its potential mechanisms. To address this, we utilize an MCD diet-induced HHcy mouse model and employ an integrative methodological framework encompassing cardiac ultrasonography, histopathological analysis, serum biochemical assays, 16S rRNA gene sequencing, and untargeted metabolomics. The findings of this research are intended to contribute to the development of novel microbiome-based intervention strategies for the prevention and treatment of HHcy-associated cardiac injury, thereby providing both experimental evidence and theoretical insights.

## 2. Methods

### 2.1. Strain Screening

To isolate *L. plantarum* P133, the probiotic strain capable of folate production, a total of 126 samples were collected from various sources, including traditional fermented foods, fresh vegetables and fruits, and colostrum. The isolation procedure was as follows: solid samples were finely chopped and homogenized, whereas liquid samples were directly inoculated into test tubes containing 5 mL of de Man, Rogosa and Sharpe (MRS) liquid medium. Following vortex mixing, the cultures were anaerobically incubated at 37 °C for 24 to 36 h to enrich bacterial growth. Subsequently, the enriched cultures were serially diluted and plated onto calcium carbonate-supplemented MRS solid medium, then incubated anaerobically at 37 °C for 24 h. Based on colony morphology characteristics—such as size, color, edge regularity, and elevation—and growth rate, 3 to 6 single colonies were selected and purified through repeated streak plating, performed three times. This process yielded a total of 240 isolated and purified strains (Table S1). These strains were then inoculated into folate-depleted medium and subjected to seven consecutive passages, resulting in 149 strains demonstrating robust growth (Table S2). An initial screening for acid and bile salt tolerance, as well as antioxidant capacity, was conducted on these 149 strains, ultimately narrowing the selection to 60 candidate strains (Table S3). Quantitative analysis of folate production was performed on these candidates using high-performance liquid chromatography (HPLC) (Table S4). Seven strains exhibiting the highest folate production were selected for molecular biological identification, followed by comprehensive probiotic and safety assessments (Table S5). The target strain, *L. plantarum* P133, was identified, producing folate at a concentration of 2.11 μg/mL (Table S4), which is consistent with the fact that the whole genome results of *L. plantarum* P133 contain the cluster of genes for folate biosynthesis (Figure S1, Table S6).

### 2.2. Model Construction and Experimental Design

Male C57BL/6 mice aged six to eight weeks were obtained from Hangzhou Ziyuan and acclimated for one week prior to experimentation. The mice were then randomly assigned to four experimental groups (*n* = 8):

Normal Diet Group (Group C): Mice were maintained on a normal diet and administered 1% phosphate-buffered saline (PBS) by gastric lavage, with the volume matched to that of the folic acid gavage.

Hyperhomocysteinemia Group (Group M): HHcy was induced following established protocols. Mice in this group were fed an MCD diet and received 1% PBS by gavage, a volume equivalent to the folic acid administration [11,23].

P133 Group (Group S): Mice were fed an MCD diet and concurrently gavaged with *L. plantarum* P133 at a concentration of 10^9^ CFU/mL, with the gavage volume matched to that of folic acid administration.

Folic acid Group (Group Y): Mice were fed an MCD diet and administered folic acid at a human-relevant dose of 0.8 mg/kg via gavage, based on dosages reported in the literature [24,25].

The experimental period lasted 28 days. On the final day, all mice were fasted for 12 h prior to euthanasia. Subsequently, the colon, liver, heart, and blood were rapidly harvested for further analyses. All animal experiments were approved by the Animal Ethics Committee of Nanchang Royo Biotech Co., Ltd. (Nanchang, China) and conducted in accordance with institutional ethical guidelines (Approval No. RYE2025071801).

### 2.3. Assessment of Phenotypic Cardiac Damage

Cardiac function was assessed via transthoracic echocardiography prior to euthanasia. To enhance acoustic coupling, the thoracic hair of each mouse was carefully removed. Animals were anesthetized using 1.5% isoflurane inhalation and positioned supine on a temperature-controlled platform. Anesthesia was maintained at 1.0–1.5% isoflurane to sustain a heart rate of at least 400 beats per minute. Pre-warmed ultrasound gel was applied to the chest region to facilitate imaging. Echocardiographic data were acquired using a FUJIFILM VisualSonics Vevo F2 high-resolution ultrasound system (Toronto, ON, Canada). Left ventricular systolic and diastolic parameters were recorded employing M-mode and Doppler modalities. All images were subsequently analyzed offline utilizing Vevo LAB v5.7.0 software.

### 2.4. 16S rRNA Gene Sequencing

At the conclusion of the experimental protocol, fresh fecal specimens were aseptically collected from each mouse (*n* = 6). Samples were immediately flash-frozen in liquid nitrogen and stored at −80 °C until further processing. Amplicon libraries were prepared and sequenced on an Illumina MiSeq platform (2 × 300 bp) by Cosmos Wisdom Technologies Co., Ltd. (Hangzhou, China). Bioinformatic analyses, including quality filtering, amplicon sequence variants (ASVs) clustering, and taxonomic assignment, were performed using QIIME2 (version 2024.5) in conjunction with the SILVA database (version 138). The plateau observed in the tail of the observed features index rarefaction curve confirmed that the sequencing depth was adequate for subsequent analyses (Figure S2A).

### 2.5. Untargeted Metabolomic Profiling

The collected serum samples were entrusted to Osmos Wisdom Technologies Co., Ltd. (Hangzhou, China) for metabolite extraction and identification (*n* = 6). The instrument platform is the UHPLC-Triple TOF system of ABSCIEX Company. The chromatographic conditions were as follows: column, Waters ACQUITY BEH C18 (1.7 µm × 2.1 mm × 100 mm); mobile phase A, 0.1% formic acid in water; mobile phase B, 0.1% formic acid in acetonitrile/methanol (40/60, *v*/*v*). The column temperature was set at 40 °C. Samples were analyzed using a Q Exactive HF-X (Thermo Fisher Scientific, Bremen, Germany) mass spectrometer equipped with a heated electrospray ionization (HESI) source. Data were collected separately in both positive and negative ion modes using Full scan/ddMS2 (DDA) to obtain MS1 precursor ions for quantification and MS2 fragmentation spectra for metabolite identification. The Full Scan parameters were set as follows: resolution of 60,000, AGC target of 4 × 10^6^, maximum injection time of 100 ms, and a scan range from 60 to 900 *m*/*z*. For Full scan/ddMS2 (DDA), the top 20 MS/MS spectra (dd-MS2) were acquired at a resolution of 15,000 with an AGC target of 2 × 10^5^, maximum injection time of 25 ms, and normalized collision energies (NCE) set to stepped values of 10, 40, and 80 volts. Quality control samples (QC) are prepared by mixing the extracts of all samples in equal volumes. The volume of each QC is the same as that of the sample. They are processed and detected in the same way as the analysis samples. During the instrumental analysis process, one QC sample is inserted into every 5 to 15 analysis sample to examine the stability of the entire detection process.

Peak picking, alignment, retention time correction and feature filtering of the original data were performed using XCMS (v3.14) and Spectra R software packages (1.14.1). Metabolite annotations were accomplished using online databases (MoNA, GNPS, HMDB and MS-dial LIpidBlast databases, Level2) and internal databases (in-house database, Level1).

### 2.6. Histological Examination

Freshly harvested mouse intestinal, hepatic, and cardiac tissue specimens were fixed in 4% paraformaldehyde solution. Post-fixation, the tissues were subjected to a graded ethanol dehydration series. Subsequently, the dehydrated samples were embedded in paraffin and sectioned into slices approximately 4 μm in thickness. All paraffin-embedded sections were stained with hematoxylin and eosin (H&E) to evaluate general tissue architecture and identify pathological alterations. Furthermore, intestinal tissue sections underwent Alcian Blue-Periodic Acid-Schiff (AB-PAS) staining to specifically detect mucopolysaccharides within the intestinal mucosa. Following staining procedures, the sections were examined using a Nikon Ti-U-100 microscope (Nikon, Tokyo, Japan) to assess histopathological changes.

### 2.7. Serum Biochemical Analysis and Organ Function Evaluation

Serum levels of aspartate aminotransferase (AST), nitric oxide (NO), and lactate dehydrogenase (LDH) were quantified utilizing commercial assay kits procured from Nanjing Jiancheng Bioengineering Institute (Nanjing, China). Additionally, concentrations of folate (FA), homocysteine (Hcy), cardiac troponin I (Ctni), and cardiac troponin T (Ctnt) were determined via enzyme-linked immunosorbent assay (ELISA) kits supplied by Shanghai Yansheng Industrial Co., Ltd. (Shanghai, China). All assays were conducted in strict accordance with the manufacturers’ protocols.

To evaluate oxidative stress within the cardiac tissues, enzymatic activities and levels of superoxide dismutase (SOD), malondialdehyde (MDA), catalase (CAT), and glutathione (GSH) were measured. Furthermore, hepatic levels of S-adenosylmethionine (SAM) and S-adenosylhomocysteine (SAH) were quantified utilizing kits from Nanjing Jiancheng Bioengineering Institute, following the manufacturer’s instructions.

### 2.8. Statistical Analysis

All statistical analyses were conducted utilizing GraphPad Prism version 9.1.0 (GraphPad Software, San Diego, CA, USA), with additional significance testing performed using SPSS version 22.0 (IBM Corp., Armonk, NY, USA). Normality was assessed using the Shapiro–Wilk test. Homogeneity of variances was tested by Levene’s test. Group comparisons were carried out using one-way analysis of variance (ANOVA), followed by appropriate post hoc tests (Tukey’s HSD or Dunnett’s T3) where applicable. Moreover, the FDR (Benjamini–Hochberg) correction was applied for microbiome and metabolomics data.

## 3. Results

### 3.1. Probiotic Characterization of L. plantarum P133

**Figure 1:**
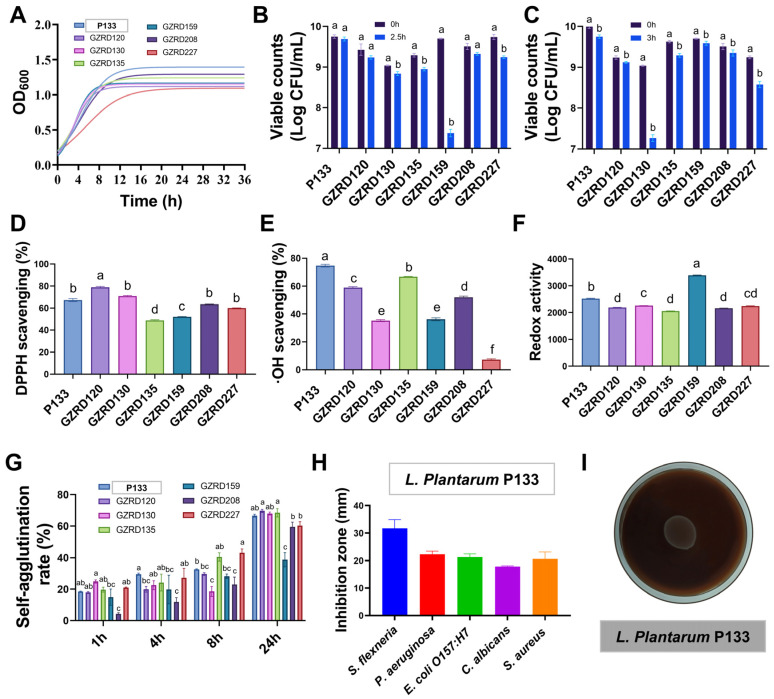
Assessment of the in vitro probiotic characteristics of *L. plantarum* P133. (**A**) The growth curve was determined by measuring optical density; Test of (**B**) acid tolerance and (**C**) bile salt tolerance; (**D**–**F**) Antioxidant capacity test, including DPPH scavenging rate, ·OH scavenging rate, and Redox activity; (**G**) Self-agglutination rate and (**H**) Antibacterial activity test of *L. plantarum* P133; (**I**) Hemolysis assay of *L. plantarum* P133. (The experiments were repeated at least three times. One-way ANOVA with Tukey’s post hoc test. Unpaired two-tailed Student’s *t*-test. Data represented as means ± SD. Different lower-case letters represent significant differences between groups. *p* < 0.05). *L. plantarum* P133: *Lactiplantibacillus plantarum* P133.

Growth curve analysis revealed that *L. plantarum* P133 entered the logarithmic phase of growth at 2 h under culture conditions maintained at 37 °C, reaching maximal colony density at 18 h (Figure 1A). Then, we comprehensively assessed its probiotic potential. Acid and bile salt tolerance tests indicated robust survivability, as the strain maintained a viable count exceeding 10^9^ CFU/mL after 2.5 h in a medium at pH 3 and 3 h in a medium containing 0.3% bile salts (Figure 1B,C). Antioxidant assays demonstrated that *L. plantarum* P133 possesses substantial antioxidant capabilities, with a DPPH radical scavenging rate of 67.19 ± 1.36%, a hydroxyl radical (•OH) scavenging rate of 74.63 ± 1.02%, and a reducing power equivalent to 2522 ± 11.49 μmol/L L-ascorbic acid (Figure 1D–F). The self-agglutination assay showed increasing self-agglutination rates of 18.43 ± 0.36%, 29.55 ± 0.71%, 32.61 ± 0.34%, and 66.64 ± 0.93% at 1, 4, 8, and 24 h of incubation, respectively, suggesting strong colonization potential of the intestinal mucosa (Figure 1G). Antimicrobial testing revealed significant inhibitory activity against various pathogenic microorganisms, with inhibition zone diameters (mm) measured as follows: *Shigella flexneri* (31.67 ± 3.21), *Pseudomonas aeruginosa* (22.33 ± 1.15), *Escherichia coli O157* (21.33 ± 1.15), *Candida albicans* (17.83 ± 0.29), and *Staphylococcus aureus* (20.67 ± 2.52) (Figure 1H). Hemolysis assays confirmed the absence of notable hemolytic activity, indicating favorable biosafety for potential in vivo applications (Figure 1I). Genomic mining further revealed an extensive repertoire of carbohydrate-active enzymes (CAZymes) and secondary metabolite biosynthesis gene clusters. Specifically, the P133 genome encodes 115 CAZymes, including 59 glycoside hydrolases (GHs) (Table S7). The high GH count suggests a strong capacity to degrade diverse dietary fibers, potentially producing SCFAs and fermentable sugars that may cross-feed beneficial gut bacteria. In addition, 134 biosynthetic gene clusters were identified, comprising 41 NRPS, 40 T3PKS, 20 terpenes, 19 cyclic-lactone-autoinducers, and 14 RiPP-like clusters (Table S8). These clusters are implicated in the production of antimicrobial peptides, immunomodulatory compounds, and quorum-sensing molecules, which may contribute to the strain’s in vitro antimicrobial activity and in vivo gut microbiota modulation. Alignment against VFDB and CARD (identity > 80%, coverage > 80%, E-value ≤ 1 × 10^−5^) revealed no known virulence or resistance genes, supporting the safety of P133 for food/pharmaceutical applications. Collectively, these results suggest that the isolated *L. plantarum* P133 strain not only exhibits excellent folate biosynthesis capacity but also demonstrates promising probiotic attributes.

### 3.2. Protective Effects of L. plantarum P133 on Cardiac Function in Murine Models of Hyperhomocysteinemia

**Figure 2:**
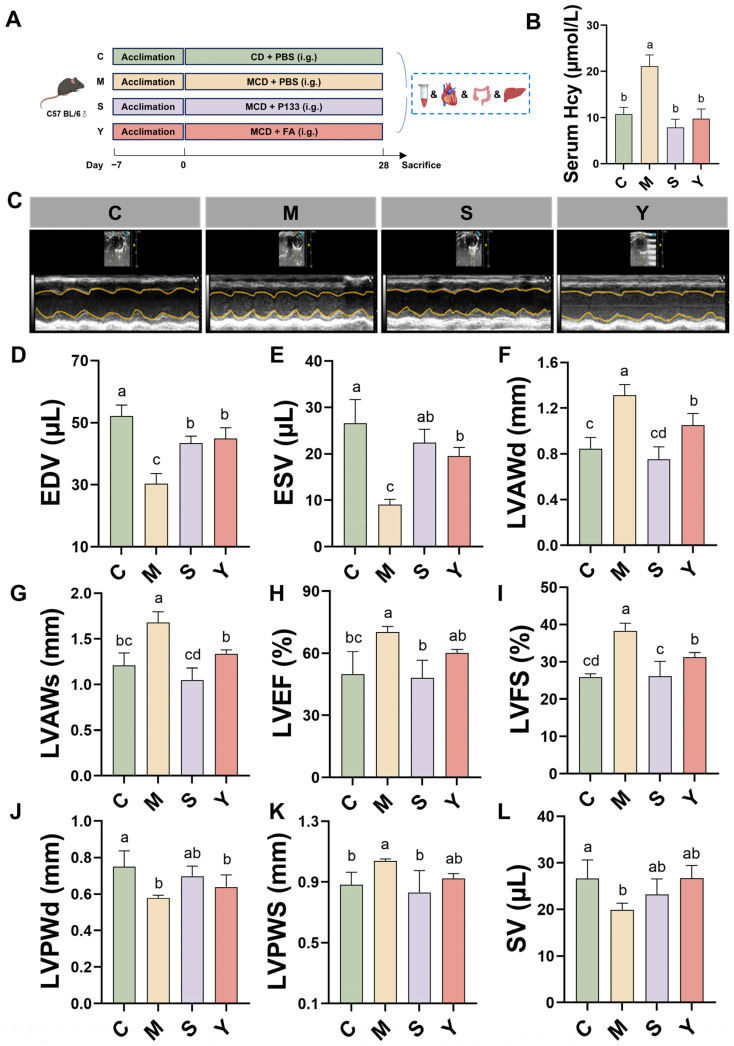
Echocardiographic imaging of mice. (**A**) Diagram of animal experiment design. (**B**) Hcy levels in serum. (**C**) Representative echocardiographic images. The (**D**) EDV, (**E**) ESV, (**F**) LVAWd, (**G**) LVAWs, (**H**) LVEF, (**I**) LVFS, (**J**) LVPWd, (**K**) LVPWs, and (**L**) SV of the mouse heart. (*n* = 6–7. Data represented as means ± SD. One-way ANOVA with Tukey’s post hoc test. Different lower-case letters represent significant differences between groups. *p* < 0.05). Hcy: homocysteine. EDV: End-Diastolic Volume. ESV: End-Systolic Volume. LVAWd: Left Ventricular Anterior Wall thickness at end-diastole. LVAWs: Left Ventricular Anterior Wall thickness at end-systole. LVEF: left ventricular ejection fraction. LVFS: left ventricular shortening fraction. LVPWd: Left Ventricular Posterior Wall thickness at end-diastole. LVPWs: Left Ventricular Posterior Wall thickness at end-systole. SV: Stroke Volume.

During the 28-day experimental period, the serum Hcy content of mice in group M increased significantly (Figure 2A,B). Following treatment with *L. plantarum* P133 or folic acid, serum Hcy levels in all mice approached the normal range, indicating that both interventions alleviated MCD diet-induced HHcy. The heart is a key target organ in HHcy pathology. Echocardiographic imaging demonstrated (Figure 2C–L) that, relative to the control group, mice in the model group exhibited significantly decreased ESV, EDV, and SV, alongside elevated LVFS and LVEF. Additionally, measurements revealed marked increases in LVAWd, LVAWds, and LVPWs during both diastole and systole. However, no significant difference in LVPWd was found among the four groups. Treatment with *L. plantarum* P133 or folic acid normalized these parameters, indicating that both interventions mitigate MCD diet-induced compensatory cardiac remodeling.

**Figure 3:**
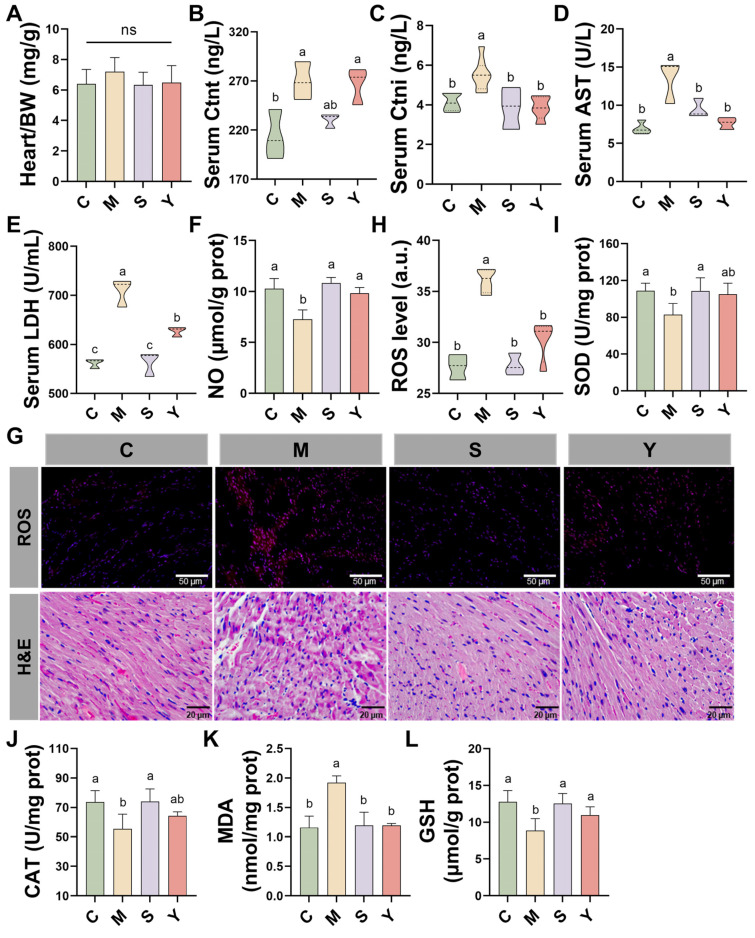
Effects of *L. plantarum* P133 on cardiac injury in mice. (**A**) Heart index. Determination of (**B**) Ctnt activity, (**C**) Ctni activity, (**D**) AST activity, and (**E**) LDH activity in serum. (**F**) NO content in mouse hearts. (**G**) Cardiac images with H&E staining, representative confocal microscopic images of ROS, and (**H**) the quantitative results. (**I**) Cardiac SOD activity. (**J**) Cardiac CAT activity. The content of (**K**) MDA and (**L**) GSH. (*n*  =  6–7. Data represented as means ± SD. One-way ANOVA with Tukey’s post hoc test. Different lower-case letters represent significant differences between groups. *p* < 0.05). Ctnt: cardiac troponin T. Ctni: cardiac troponin I. AST: aspartate aminotransferase. LDH: lactate dehydrogenase. NO: nitric oxide. ROS: reactive oxygen species. ns: not significant (*p* ≥ 0.05). SOD: superoxide dismutase. MDA: malondialdehyde. CAT: catalase. GSH: glutathione. Dashed lines in violin plots: indicate the median value of each group.

As shown in Figure 3A, there was no significant statistical difference in the cardiac organ coefficients among the four groups of mice. Compared with the control group, group M exhibited significantly elevated serum levels of Ctnt, Ctni, AST and LDH, while cardiac NO content was significantly reduced. Moreover, group S showed a more pronounced ameliorative effect than group Y, particularly for Ctnt and LDH. Cardiac histopathological analysis visually revealed the cardiac dysfunction resulting from MCD diet-induced HHcy and the alleviating effects of P133 and folic acid. H&E-stained sections showed that hearts from MCD-fed mice exhibited substantial structural abnormalities, including disorganized myocardial fibers, disrupted myocyte architecture, and marked inflammatory cell infiltration. These abnormalities were significantly attenuated in mice administered *L. plantarum* P133 or folic acid. Specifically, group Y displayed minor residual myofibrillar disorganization, whereas group S showed myocardial tissue integrity and a regular myofibrillar pattern closely resembling that of the control group C.

Oxidative stress induction and redox imbalance in vivo are recognized as critical toxicological mechanisms underlying diet-induced cardiac injury. Consequently, oxidative stress parameters were assessed. As illustrated in Figure 3G–L, treatment with either *L. plantarum* P133 or folic acid ameliorated these oxidative stress markers in HHcy mice induced by MCD diet to varying degrees. Notably, *L. plantarum* P133 more effectively restored SOD and CAT activities and GSH concentrations, concurrently reducing MDA levels, thereby normalizing the cardiac redox balance. These results suggest that both *L. plantarum* P133 and folic acid confer substantial cardioprotective effects in MCD diet-induced HHcy mouse models by modulating compensatory cardiac remodeling, attenuating cardiomyocyte injury, enhancing endothelial function, and mitigating oxidative stress.

### 3.3. Effects of L. plantarum P133 on Mice with Hyperhomocysteinemia

**Figure 4:**
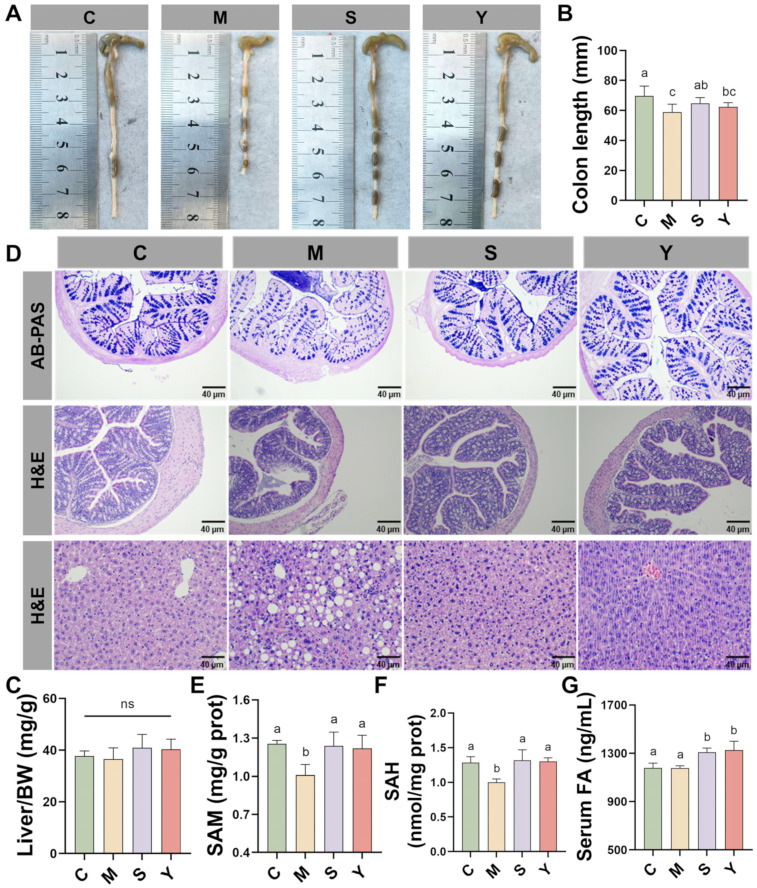
(**A**) Representative colon images and (**B**) the quantitative results. (**C**) Liver index. (**D**) Colonic images with AB-PAS staining, H&E staining, and hepatic images with H&E staining. The content of (**E**) SAM and (**F**) SAH in the liver. (**G**) FA levels in serum. (*n* = 6–7. Data represented as means ± SD. One-way ANOVA with Tukey’s post hoc test. Different lower-case letters represent significant differences between groups. *p* < 0.05). SAM: S-adenosylmethionine. SAH: S-adenosylhomocysteine. FA: folate. ns: not significant (*p* ≥ 0.05).

Mice in group M exhibited a statistically significant reduction in colon length compared to controls (Figure 4A,B), suggestive of chronic inflammatory processes within colonic tissues. Although group Y showed a non-significant trend toward increased colon length, group S effectively mitigated MCD diet-induced colon shortening. Histopathological analyses corroborated these observations; AB-PAS and H&E staining revealed that the MCD diet caused mucus depletion, inflammatory cell infiltration, mild submucosal edema, and localized muscularis edema (Figure 4C,D). Treatment with either *L. plantarum* P133 or folic acid ameliorated these intestinal lesions to varying extents, with the P133-treated group exhibiting superior reparative outcomes relative to the folic acid-treated group. These results suggest that *L. plantarum* P133 and folic acid offer significant protection to the intestinal epithelial barrier, with *L. plantarum* P133 demonstrating enhanced efficacy.

H&E staining visualizes tissue structure. In contrast to the well-organized hepatic architecture observed in Group C, livers from Group M exhibited periportal inflammatory infiltration, disorganized hepatocyte arrangement and numerous intercellular vacuoles, which are indicative of severe steatosis. The administration of *L. plantarum* P133 or folic acid markedly attenuated hepatic steatosis and tissue damage. Interestingly, the folic acid-treated group displayed mild hepatocyte disorganization and nuclear condensation, whereas the P133-treated group exhibited hepatic histology that most closely resembled that of the control group (Figure 4D). Consistent with the changes in liver tissue structure, treatment with *L. plantarum* P133 or folic acid restored SAM, SAH and FA levels in MCD-induced HHcy mice, further supporting their protective effect on liver metabolism (Figure 4E–G). Taken together, these findings demonstrate that *L. plantarum* P133 substantially mitigates MCD-induced intestinal and hepatic injury in HHcy mice, modulating disrupted hepatic methylation metabolism and exhibiting superior protective efficacy compared to folic acid.

### 3.4. L. plantarum P133 Altered Gut Microbiota Composition in Mice with Hyperhomocysteinemia

Given that mice with MCD diet-induced HHcy exhibit both intestinal and cardiac toxicity, this study utilized high-throughput 16S rRNA gene sequencing to perform a comprehensive analysis of fecal microbiota. This approach aimed to evaluate the modulatory effects of *L. plantarum* P133 on MCD diet-induced alterations in gut microbial communities.

**Figure 5:**
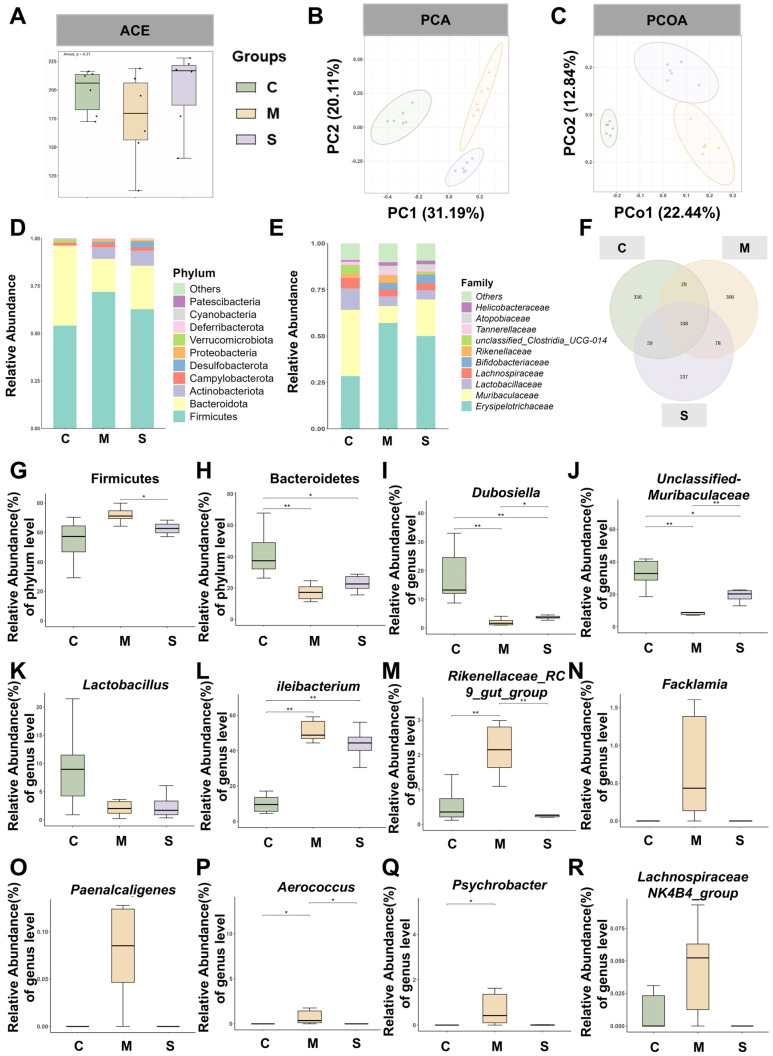
*L. plantarum* P133 altered gut microbiota composition in mice with hyperhomocysteinemia. (**A**) ACE indexes of microbial α diversity. (**B**,**C**) PCA and PCoA analysis of microbial β diversity. (**D**,**E**) The community barplot diagram of the microbiome at the phylum and family levels. (**F**) The Venn diagram depicted the overlap of ASVs within the microbiota. (**G**,**H**) Relative abundance of specific phyla, including Firmicutes and Bacteroidetes. (**I**–**R**) Relative abundance of specific genus, including *Dubosiella*, *Unclassified-Muribaculaceae*, *Lactobacillus*, *Ileibacterium*, *Rikenellaceae_RC9_gut_group*, *Facklamia*, *Paenalcaligenes*, *Aerococcus*, *Psychrobacter*, and *Lachnospiraceae NK4B4_group*. (*n* = 6. Data represented as means ± SD. One-way ANOVA with Tukey’s post hoc test. * *p* < 0.05, ** *p* < 0.01). ACE: Abundance-based Coverage Estimator. PCA: principal component analysis. PCoA: principal coordinates analysis.

Although α-diversity metrics, specifically the ACE index reflecting community richness, did not reveal statistically significant differences among experimental groups, PCA and PCoA based on Bray–Curtis dissimilarity demonstrated distinct microbial community structures between group M and group C (Figure 5A–C). This finding indicates that the MCD diet substantially altered the β-diversity of the gut microbiota in mice. Following intervention with *L. plantarum* P133, the disrupted microbial community structure in group M was partially restored, exhibiting increased similarity to that of group C. These findings suggest that although certain microbial taxa are common across groups, their relative abundances and compositional patterns differ significantly. Thus, the microbiota’s compositional structure becomes increasingly discernible at finer taxonomic resolutions.

Specifically, compared with the control group, MCD dietary exposure significantly increased the abundance of the phylum Bacteroidetes while reducing that of Firmicutes. Correspondingly, the abundance of families Erysipelotrichaceae, Rikenellaceae, Aerococcaceae, and Tannerellaceae increased, whereas the abundance of Muribaculaceae, Lachnospiraceae, Sutterellaceae, Ruminococcaceae, and Lactobacillaceae decreased significantly in group M relative to group C. At the genus level, bacterial genera exhibited significantly altered abundances in the intestinal samples of group M compared with the control group. Among these, *Ileibacterium*, *Rikenellaceae_RC9_gut_group*, *Facklamia*, *Paenalcaligenes*, *Aerococcus*, *Psychrobacter*, and *Lachnospiraceae_NK4B4_group* showed increased abundance, whereas *Dubosiella*, unclassified *Muribaculaceae*, and *Lactobacillus* showed decreased abundance (Figure 5D–R and Figure S2B,C).

### 3.5. L. plantarum P133 Modulated Serum Metabolites Composition in Mice with Hyperhomocysteinemia

**Figure 6:**
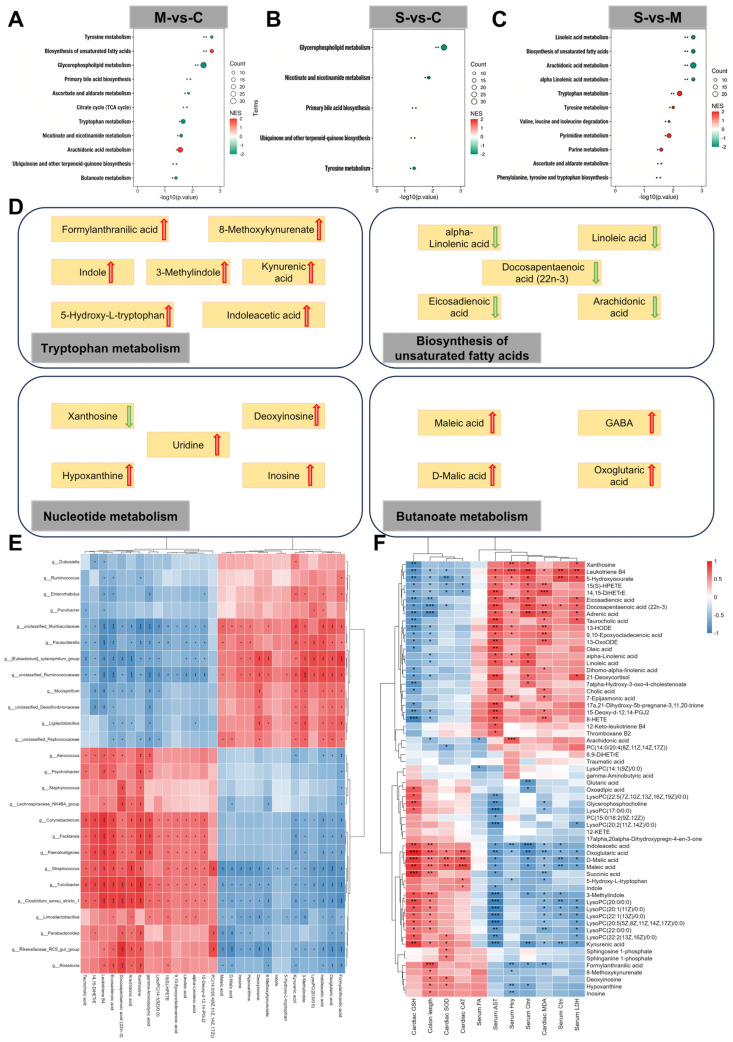
*L. plantarum* P133 modulated serum metabolites composition in mice with hyperhomocysteinemia. MSEA analysis of differential metabolites between (**A**) the M and C groups, (**B**) the S and C groups, and (**C**) the S and M groups. (**D**) Diagram of serum metabolic alteration. (**E**) Correlation heatmap analysis of microbiota and metabolite parameters using Spearman’s rank correlation. (**F**) Correlation heatmap analysis of metabolite parameters and phenotypic damage indices using Spearman’s rank correlation. (*n* = 6. Data represented as means ± SD. One-way ANOVA with Tukey’s post hoc test. FDR correction (q < 0.05) was applied. The red arrow indicates a significant increase, and the green arrow indicates a significant decrease. * *p* < 0.05, ** *p* < 0.01, *** *p* < 0.001).

To better understand the potential mechanism by which *L. plantarum* P133 alleviates MCD diet-induced cardiotoxicity in HHcy mice, untargeted metabolomics profiling was performed to identify relevant metabolites. Based on the differential metabolite regulatory network derived from MSEA enrichment outcomes and the heatmap of significantly altered metabolites, the metabolites differing between the two groups predominantly clustered within the amino acid, nucleotide, and fatty acid categories. These metabolites chiefly influenced KEGG pathways such as butyrate metabolism, tryptophan metabolism, biosynthesis of unsaturated fatty acids, linoleic acid metabolism, arachidonic acid metabolism, purine metabolism, and pyrimidine metabolism (Figure 6A–C). To elucidate metabolic biomarkers underlying the cardioprotective effects of *L. plantarum* P133 in HHcy mice, differential metabolites between the S and M groups were further examined, alongside potential modifications induced by folate synthesized by *L. plantarum* P133. Compared with group M, the levels of multiple key metabolites in group S were significantly upregulated, further demonstrating the intervention and protective effect of *L. plantarum* P133, specifically including: tryptophan metabolization-related products (indoleacetic acid, 8-methoxy-kynurenate, indole, kynurenine, 5-hydroxy-L-tryptophan, 3-methylindole, formylo-aminobenzoic acid); Nucleotide metabolism-related products (deoxyinosine, inosine, hypoxanthine, etc.); And γ-aminobutyric acid (GABA), α-ketoglutaric acid, etc. (Figure 6D).

Subsequently, Spearman’s correlation analysis was conducted to examine the associations between the composition of gut microbiota groups and their metabolic profiles (Figure 6E). Heatmaps illustrating metabolic associations demonstrated both significantly positive and negative correlations between differential metabolite levels and the identified bacterial taxa. Notably, dominant genera within Group S, including *Dubosiella* and *unclassified-Muribaculaceae*, exhibited significant positive correlations with tryptophan and nucleotide metabolites, alongside significant negative correlations with linoleic acid metabolites. Conversely, dominant genera in Group M, such as *Rikenellaceae_RC9_gut_group*, *Facklamia*, *Paenalcaligenes*, *Aerococcus*, *Psychrobacter*, and the *Lachnospiraceae NK4B4_group*, displayed inverse correlation patterns. In summary, the current study provides evidence that *L. plantarum* P133 represents a safe and effective probiotic strategy for the treatment of cardiac injury induced by HHcy, thereby offering partial validation of its potential application in preclinical mouse models.

## 4. Discussion

HHcy is an independent risk factor for cardiovascular diseases and can damage the functions of organs such as the heart through multiple pathways, posing a significant threat to the health of the population. Given the limited effectiveness of traditional intervention programs in complex metabolic disorders and the increasing evidence supporting probiotic intervention in the treatment of metabolic diseases, this study explored the potential protective effect of administration of *L. plantarum* P133 on the hearts of HHcy mice and proposed novel insights into its possible systemic mechanisms.

Previous studies have shown that *L. plantarum* can mitigate HHcy by secreting bioactive metabolites such as butyrate and modulating gut microbiota composition [26,27,28,29]. Folate, as a classical therapeutic agent, directly participates in the remethylation cycle of homocysteine, thereby effectively lowering its systemic levels [22]. Huang et al. further elucidated that folate alleviates complications associated with HHcy via intricate cellular mechanisms [30]. For example, in idiopathic pulmonary fibrosis, folate reduces homocysteine-induced homocysteinylation of the autophagy-related protein STX17, thereby restoring autophagic flux and attenuating fibrotic progression [30]. Based on these findings, we hypothesize that *L. plantarum* P133 may confer protective effects through dual mechanisms: firstly, by in situ folate synthesis replenishing methyl donors, and secondly, by modulating gut microbiota and their metabolites to systemically ameliorate the metabolic milieu. These synergistic pathways may offer a multifaceted strategy for preventing and treating HHcy and its associated multi-organ pathologies.

To investigate the impact of *L. plantarum* P133 on health parameters in HHcy, a murine model was established through administration of an MCD diet. Validation of the HHcy model was achieved by quantifying serum Hcy concentrations and referencing established international diagnostic criteria alongside prior empirical data (Figure 2B) [23,31]. Numerous epidemiological and clinical investigations have established that elevated plasma Hcy concentrations constitute an independent risk factor for cardiovascular pathologies, which continues to represent a predominant cause of mortality on a global scale [6,32]. The heart, as the primary organ implicated in these conditions, exhibits structural and functional alterations closely associated with the onset and progression of cardiovascular disorders. Our study employed echocardiography, serum biochemical assays, histopathological evaluation, and oxidative stress analyses to examine the potential associations of *L. plantarum* P133 with cardiac parameters in mice subjected to HHcy induced by an MCD diet.

A study conducted on wild-type mice demonstrated that following eight weeks of treadmill training, there was an increase in left ventricular mass without alteration in chamber diameter, accompanied by a reduction in ESV, which consequently resulted in elevated EF and FS [33]. Conversely, during the initial phase of sepsis, the heart may sustain circulatory function by augmenting contractility and decreasing chamber volume. However, this “high-functioning state” arises in the context of underlying myocardial cellular injury [34]. It has been described as a compensatory mechanism that precedes cardiac decompensation or may be directly induced by pathological processes such as severe edema or inflammatory infiltration. The alterations observed in cardiac parameters of Group M appear to be consistent with an early compensatory response, characterized by enhanced myocardial contractility and ventricular wall thickening. Nevertheless, without direct pressure-volume loop analysis or fibrosis quantification, we cannot definitively determine whether this hyperdynamic state represents adaptive compensation or an early stage of maladaptive remodeling [35,36].

Given the critical role of cardiac contractile function in maintaining cardiovascular health, sustained contractile abnormalities may exacerbate disease progression. Ctnt and Ctni, integral components of the myosin-troponin complex in cardiomyocytes, regulate calcium-mediated myocardial contraction and are typically confined intracellularly under physiological conditions [37]. AST and LDH are intracellular enzymes present at minimal levels in serum. The observed significant elevations in serum concentrations of these four biomarkers in the model group are consistent with myocardial cell injury and necrosis (Figure 3A–E) [38]. NO, a pivotal signaling molecule, maintains vascular endothelial function and myocardial homeostasis [39]. Alterations in NO levels reflect the dynamic balance of vascular function, myocardial cell status, inflammatory responses, and tissue repair processes. Cardiac tissue analysis revealed a significant reduction in NO levels in the model group, which is also consistent with impaired endothelial function and progressive myocardial damage (Figure 3F). This finding aligns with reports that HHcy inhibits endothelial nitric oxide synthase activity and increases its uncoupling [40]. Taken together, these results suggest that *L. plantarum* P133 may be associated with reduced cardiac injury in MCD-fed HHcy mice. The observed improvements correlate with changes in cardiac remodeling-related parameters, lower myocardial injury markers, and attenuated oxidative stress. Consistent with prior research, Hcy exerts cardiotoxic effects through multiple pathways, including the induction of endothelial dysfunction, oxidative stress, and chronic inflammation. Zheng et al. further elucidated underlying molecular mechanisms, demonstrating that aberrant N-homocystoylation of key signaling proteins, such as β-arrestin, disrupts cellular signaling pathways [41]. Concurrently, modifications of lysosomal V-ATPase subunits impair their function, leading to defective clearance of cellular metabolic waste and subsequent cell death [42]. Our study is consistent with the concept that folic acid supplementation facilitates homocysteine remethylation, thereby potentially reducing cardiovascular risk associated with HHcy. Moreover, the superior efficacy observed with *L. plantarum* P133 treatment warrants further investigation into its nuanced biological effects, which may involve microbiome-related pathways.

The liver and intestine are not only primary target organs affected by complications arising from HHcy, but disturbances in their homeostasis also exert profound effects on systemic metabolism, thereby exacerbating HHcy. Mice subjected to the HHcy-inducing diet exhibited significant deviations in markers indicative of organ damage and metabolic dysfunction relative to controls. Given the intestine’s pivotal role in digestion, nutrient absorption, immune defense, and barrier maintenance, its structural and functional integrity is essential for overall systemic health [43,44]. The liver, as the central organ responsible for metabolism and detoxification, undergoes both functional and structural changes that serve as critical indicators of injury in this model [45]. At the molecular level, SAM and SAH are key intermediates in hepatic methylation pathways integral to Hcy and folate metabolism. Collectively, these findings suggest that *L. plantarum* P133 is associated with the mitigation of MCD diet-induced intestinal and hepatic injury in HHcy mice and with the modulation of disrupted hepatic methylation metabolism, showing a more favorable outcome compared to folic acid. According to Ahn et al., betaine contributes to the restoration of disrupted hepatic methionine cycles and the reduction in plasma Hcy concentrations [23]. Folate supplementation, serving as an alternative methyl donor within the Hcy remethylation pathway, accounts for the normalization of methionine metabolism observed in both the S and Y groups. Notably, despite comparable or slightly elevated folate levels in the Y group, the S group demonstrated more substantial ameliorative effects. This disparity may be attributed to differences in folate form and bioavailability. Folate synthesized by lactic acid bacteria predominantly exists in the polyglutamate form, which is structurally analogous to natural folate and may confer enhanced bioavailability, whereas chemically synthesized folic acid primarily occurs as the monoglutamate form [46]. Furthermore, *L. plantarum* P133 may exert synergistic effects through multiple mechanisms, including modulation of the gut microbiota and the production of additional beneficial metabolites.

The gut microbiota serves as a critical regulator of human health, playing a pivotal role not only in nutrient digestion and host immune function but also in the pathogenesis of chronic conditions such as obesity, NAFLD, and cardiovascular disease [47]. Through 16S rRNA gene sequencing, P133 administration was associated with reversal of MCD-related intestinal microbiota dysbiosis (characterized by the reduction in Muribaculaceae, *Lachnospiraceae*, *Sutterellaceae*, *Ruminococcaceae*, *Lactobacillaceae*, and *Dubosiella*, and the increase in Erysipelotrichaceae, *Rikenellaceae*, *Tannerellaceae*, and *Aerococcus*), and these alterations were highly correlated with established biomarkers of mucus layer maintenance, inflammatory response, and cardiac injury [48,49,50,51,52,53,54,55,56]. Empirical evidence reveals that exposure to the MCD diet markedly disrupts gut microbial homeostasis, as evidenced by significant changes in β-diversity and the overall composition of the gut microbial community. It is well-established that dietary intake plays a critical role in restoring gastrointestinal homeostasis, modulating inflammation, and addressing metabolic dysfunctions [57]. Similarly, research by Wang et al. demonstrates that probiotic interventions can alleviate the detrimental effects associated with dietary challenges by modulating the gut microbiota, strengthening intestinal barrier integrity, and exerting anti-inflammatory effects [58]. These findings underscore the protective role of probiotics in maintaining gut microbial balance, aligning with the beneficial impacts of *L. plantarum* P133 observed in the present study.

Serum metabolomics directly reflects the functional state of the host and helps clarify how gut bacteria interact with host biology. This approach is invaluable for elucidating disease pathogenesis and identifying potential therapeutic targets. Notably, consistent with earlier serum folate measurements, levels of 5-methyltetrahydrofolate were much lower in group M than in group C but rose substantially after treatment with *L. plantarum* P133 (Figure S3A). This metabolite may serve as a biomarker for the mitigation of cardiac injury linked to HHcy. Folate is a key carrier in one-carbon metabolism, and its levels directly affect many methylation-dependent biochemical reactions [59]. As a major symbiotic metabolic organ, the gut microbiota strongly influences folate biosynthesis, utilization, and related metabolic networks [60]. The high-folate-producing *L. plantarum* P133 strain used here likely does more than just supply methyl groups; it may also correct the metabolic imbalances caused by HHcy by reshaping the gut microbiome and broadly adjusting core metabolic pathways that connect to one-carbon metabolism (Figure 6D and Figure S3B).

Regarding tryptophan metabolism, the S group had significantly higher levels of several intermediate substances—such as kynurenine and 5-hydroxy-L-tryptophan—than the M group in the indoleacetic acid, indole, and kynurenine pathways. These alterations have potential functional implications: indoleacetic acid, as a natural binding substance of the aryl hydrocarbon receptor (AhR), has been reported to activate the AhR pathway, which may increase the expression of intestinal tight junction proteins and potentially improve barrier function [61]. Concurrently, indole exhibits recognized anti-inflammatory and neuroprotective properties [62]. The reversal of this metabolic pattern suggests, but does not prove, *L. plantarum* P133 might enhance intestinal barrier function and systemic anti-inflammatory capacity by correcting the shift toward the harmful quinolinic acid pathway caused by folate deficiency, thus promoting the production of beneficial ferulic acid and AhR binding substances. In terms of nucleotide metabolism, the higher concentrations of key intermediates for purine and pyrimidine synthesis and repair—including inosine, deoxyinosine, hypoxanthine, and uridine—in the S group are consistent with the idea that *L. plantarum* P133 intervention helps restore the nucleic acid metabolic balance disrupted by the MCD diet. This restoration could provide essential substances for the proliferation and repair of damaged myocardial and hepatic cells [63,64]. This process is intrinsically linked to folate’s fundamental role as a one-carbon unit donor. Thus, *L. plantarum* P133 may synergistically support purine de novo synthesis by supplying folate and modulating the gut microbiota. Generally, xanthosine concentrations tend to rise simultaneously. However, the decrease observed in this study may reflect that cells undergo distinct metabolic adjustments in response to oxidative stress, energy deficiency, and reparative demands [65]. Moreover, changes in other key metabolites are consistent with multiple targets. The elevated levels of GABA may contribute to the inhibition of intestinal inflammatory factor release via its role as a neurotransmitter [66]. Additionally, the increased concentration of α-ketoglutarate, a key intermediate in the tricarboxylic acid cycle, implies that *L. plantarum* P133 might be associated with the enhancement of mitochondrial energy metabolism, which could supply energy for the repair of organs including cardiac tissue [67].

Subsequently, Spearman correlation analysis further substantiated the pivotal role of these metabolites in mediating cardioprotective effects, aligning with evidence reported in previous studies [68,69]. Taken together, these results show that administration of *L. plantarum* P133 elicited changes in the structural and compositional characteristics of the gut microbiota, which were accompanied by notable alterations in serum metabolic profiles. In summary, the current study provides evidence that *L. plantarum* P133 represents a safe and effective probiotic strategy for ameliorating cardiac injury associated with HHcy, thereby offering partial support for its potential application in preclinical mouse models.

Despite the convincing evidence, this study has several limitations. Although multi-omics methods have revealed important associations between *L. plantarum* P133, gut microbiota, serum metabolites and MCD-induced cardiac injury in HHcy mice, these correlational data cannot establish a clear causal relationship. The direct functional effects of bacteria and metabolites require verification through intervention studies, such as fecal microbiota transplantation or the use of germ-free animals. Furthermore, although we have identified systemic metabolic changes, the specific microbe-derived metabolites and their direct targets in the heart remain to be elucidated. The multiple protective effects of *L. plantarum* P133 may be driven by the synergy of folate-dependent and folate-independent systems, with interactions between these systems forming a complex regulatory network. On the one hand, folate synthesized by *L. plantarum* P133 could directly affect the core metabolic defects of HHcy; on the other hand, its probiotic properties (such as immune regulation and microbiota modulation) may independently contribute to organ protection. Future research should employ a folate-synthesis-deficient mutant of *L. plantarum* P133 for parallel in vivo and in vitro comparisons to clarify and quantify the respective and synergistic effects of the two systems in alleviating cardiac injury, thereby evaluating the strain’s dual value in ‘correcting metabolism’ and ‘regulating microecology’. In addition, mechanistic studies (e.g., FMT, gene knockout, metabolite intervention) and multiple HHcy models (e.g., a high-methionine diet or CBS-knockout mice) would be valuable to validate causality and test generalizability. Finally, long-term evaluation of P133 in complex systemic disease models should be considered to assess its chronic intervention potential.

Despite these limitations, our findings provide a strong mechanistic basis for exploring oral folate-producing *L. plantarum* P133 as a novel therapeutic strategy for HHcy and its related complications. The revealed systemic mechanisms involve the gut microbiota and metabolome, offering a potential perspective on the regulatory mechanisms of the “gut-heart axis” in cardiovascular diseases. Thus, this study provides solid strain resources and a theoretical foundation for developing new probiotic-based interventions for cardiovascular health.

## 5. Conclusions

This study confirmed that *L. plantarum* P133 exhibits strong potential for preventing cardiac injury in a mouse model of HHcy induced by an MCD diet. This is supported by echocardiographic findings, cardiac function indicators, histopathological observations, and oxidative stress analyses. Its protective effect is attributed to its sustained folate-producing capacity, as well as its ability to regulate disrupted gut microecology and host metabolic profiles. In conclusion, these results indicate that *L. plantarum* P133 represents a safe and effective probiotic intervention strategy for the prevention or management of HHcy-induced cardiac damage, thereby partially validating its potential application in mouse models.

## Data Availability

The original contributions presented in the study are included in the article/Supplementary Materials, further inquiries can be directed to the corresponding author.

## Supplementary Materials

The following supporting information can be downloaded at: https://www.mdpi.com/article/10.3390/foods15122088/s1, Figure S1: Folate biosynthesis pathway of *Lactiplantibacillus plantarum* P133; Figure S2: *Lactiplantibacillus plantarum* P133 altered gut microbiota composition in mice with Hyperhomocysteinemia; Figure S3: Metabolic Network analysis; Table S1: Strain number information table; Table S2: Table of the growth status of the strain in folate-free medium; Table S3: Growth condition tolerance table; Table S4: Strain folate production scale (μg/mL); Table S5: Molecular biological identification results of 7 LAB strains; Table S6: Folate biosynthesis-related genes; Table S7: Carbohydrate-Active enZymes; Table S8: Secondary metabolite biosynthesis.
