# Supplementary material for: Lactiplantibacillus plantarum P133, a Folate-Producing Probiotic, Ameliorates Cardiac Injury in Hyperhomocysteinemia Mice by Modulating Gut Microbiota and Serum Metabolome
Source: Foods. 2026 Jun 9;15(12):2088. doi: 10.3390/foods15122088 (PMC13298135; doi:10.3390/foods15122088)
Supplement: Supplementary file 1 [file foods-15-02088-s001.zip › foods-4331175-supplementary.pdf]

Supplementary Material:

Lactiplantibacillus plantarum P133, a folate-producing probiotic, ameliorates cardiac injury in hyperhomocysteinemia mice by modulating gut microbiota and serum metabolome

Wen Dai [1], Tiantian Jia [1], Yuanxing Wang [1], Hengyi Xu [1,2,] *

[1]State Key Laboratory of Food Science and Resources, Nanchang University, Nanchang 330047, China

[2]International Institute of Food Innovation Co., Ltd., Nanchang University, Nanchang 330200, China

* Correspondence: Hengyi Xu

State Key Laboratory of Food Science and Resources, Nanchang University

Address: 235 Nanjing East Road, Nanchang 330047, China

TEL: +86-791-8830-4447-ext-9520

FAX: +86-791-8830-4400

E-mail: hengyixu@vip.163.com

Supplementary Materials and Methods

S1.1 Determination of folate concentration in bacteria by high-performance liquid chromatography. To determine folate production, the lactic acid bacterial strain stored in 50% glycerol at −20 °C was activated and inoculated (2%, v/v) into fresh MRS broth, then incubated anaerobically at 37 °C for 16 h. After fermentation, the culture was centrifuged at 8000 r/min for 5 min. The pellet was mixed with 250 μL of chicken pancreas enzyme and incubated at 50 °C for 2 h (with constant temperature maintained). Following enzymatic hydrolysis, the mixture was centrifuged again at 8000 r/min for 5 min. The supernatant was mixed with 0.1 mL of 0.5% ammonia water, filtered through a 0.22 μm organic membrane, and stored in the dark. To prepare the standard curve, 5-methyltetrahydrofolate standard solutions of 0.4 μg/mL, 0.8 μg/mL, 1.6 μg/mL, 2.4 μg/mL, 3.2 μg/mL, and 3.6 μg/mL were used. The mobile phase consisted of methanol and phosphate-buffered saline (PBS, 0.1 mol/L, pH 7.2) at a 10:90 (v/v) ratio. The mobile phase was filtered through a 0.45 μm membrane and degassed by ultrasonication. HPLC analysis was performed on an Agilent XDB-C18 column (4.6 × 250 mm, 5 μm) with a UV detector at 280 nm, flow rate 1 mL/min, injection volume 20 μL, and run time 20 min. The results were expressed as micrograms of folate per milliliter of fermentation broth (μg/mL).

S1.2 The strains used in the experiment. The *Lactiplantibacillus plantarum* GZRD043 used in the experiment (Collection name: P133, Collection number: CCTCC M 20252434, subsequently uniformly referred to as *Lactiplantibacillus*

plantarum P133). *L. plantarum* P133 was isolated from traditional fermented pickled vegetables collected from a local market in Jilin, China.

S1.3 Whole-Genome Sequencing of *Lactiplantibacillus plantarum* P133. According to the manufacturer's instructions, genomic DNA for whole genome sequencing was extracted from P133 strain using a bacterial DNA extraction kit. Then, entrust MajorBio Biotechnology Co., Ltd. (Shanghai, China) to carry out the de novo sequencing of the bacterial genome. The sequencing experiment was based on the second-generation Illumina NovaSeq sequencing platform to complete the construction of library fragments and perform PE150 sequencing. Following data analysis was carried out on cloud.majorbio.com.

Supplementally Figures

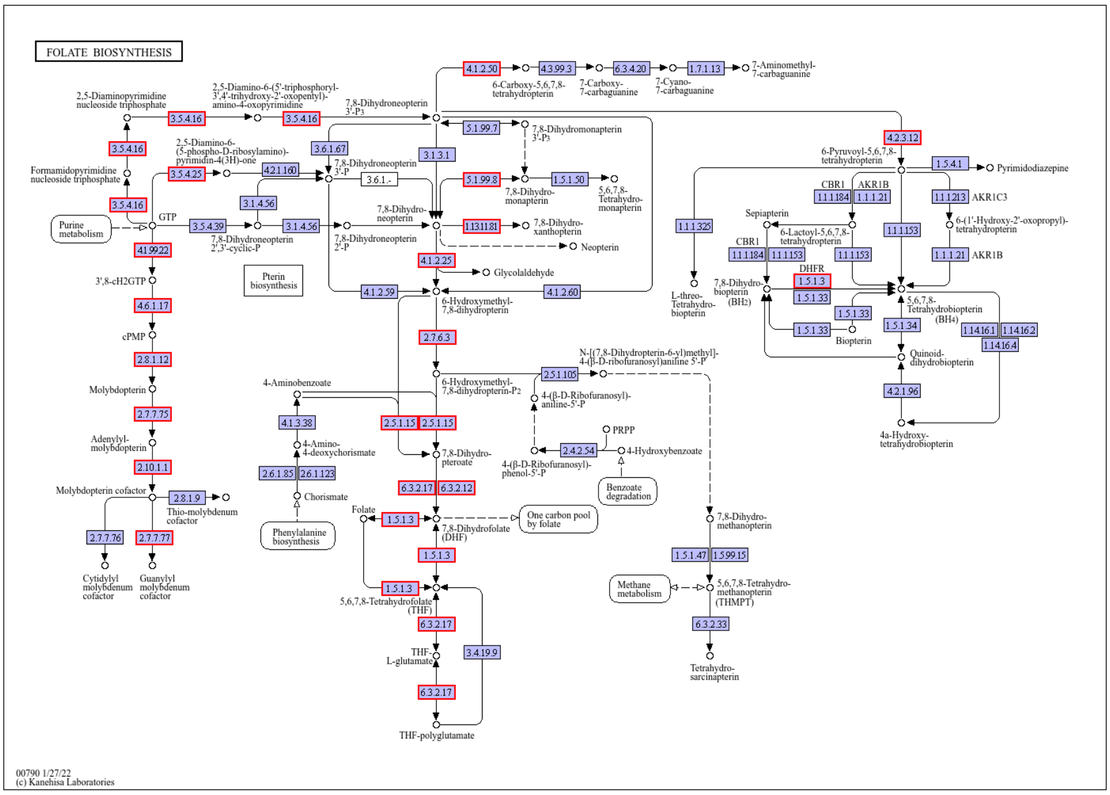

Figure S1. Folate biosynthesis pathway of *Lactiplantibacillus plantarum* P133.

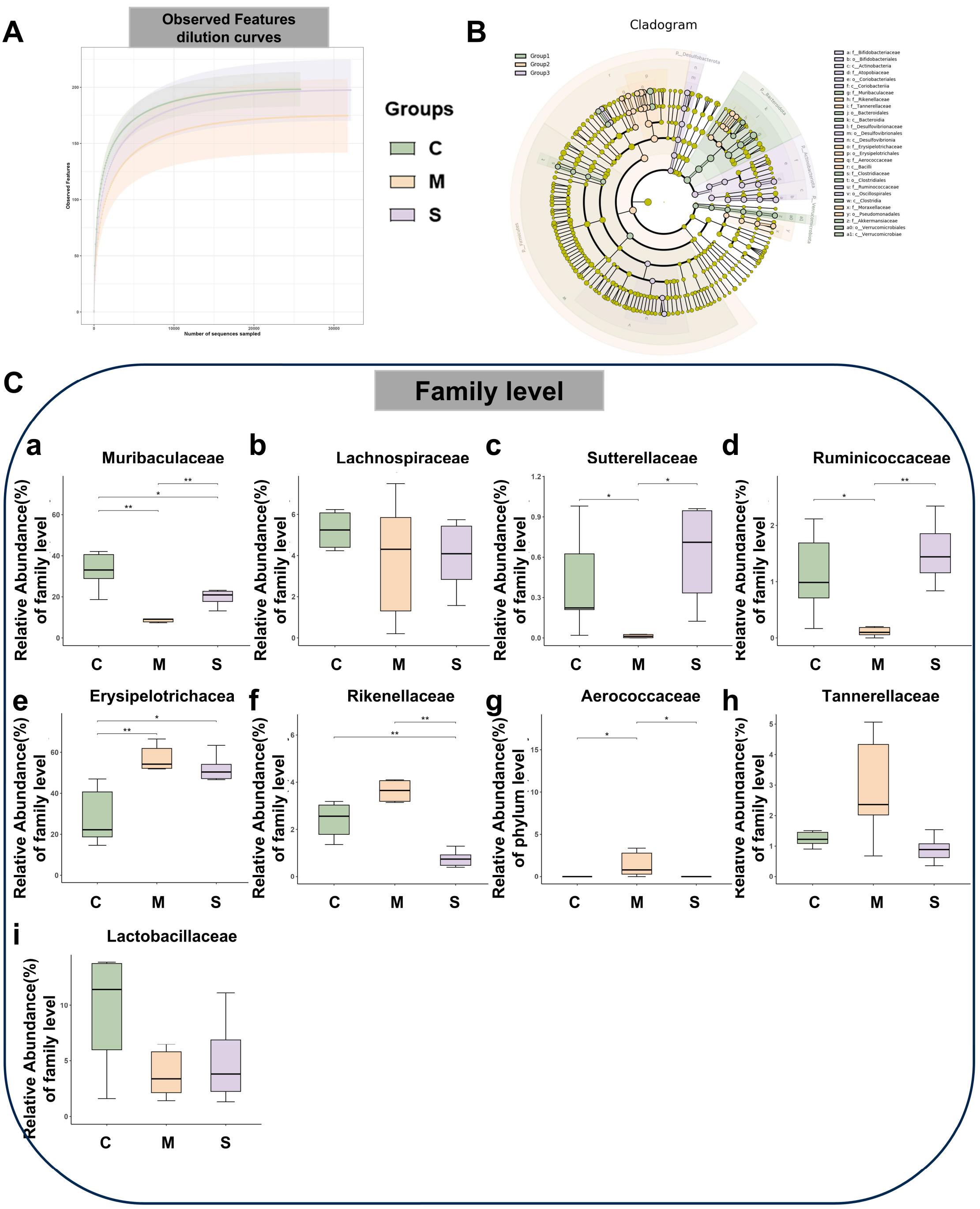

Figure S2. ***Lactiplantibacillus plantarum*** **P133 altered gut microbiota composition in mice with Hyperhomocysteinemia.** (A) Observed Features dilution curves. (B) Evolutionary branch diagram of different species. Relative abundance of specific bacteria at the family level: (C) a-i. Muribaculaceae Lachnospiraceae, Sutterellaceae, Ruminicoccaceae, Erysipelotrichaceae, Rikenellaceae, Aerococcaceae, Tannerellaceae, lactobacillaceae family. (n = 6. Data represented as means ± SD. One-way ANOVA with Tukey's post hoc test. * $p < 0.05$, ** $p < 0.01$).

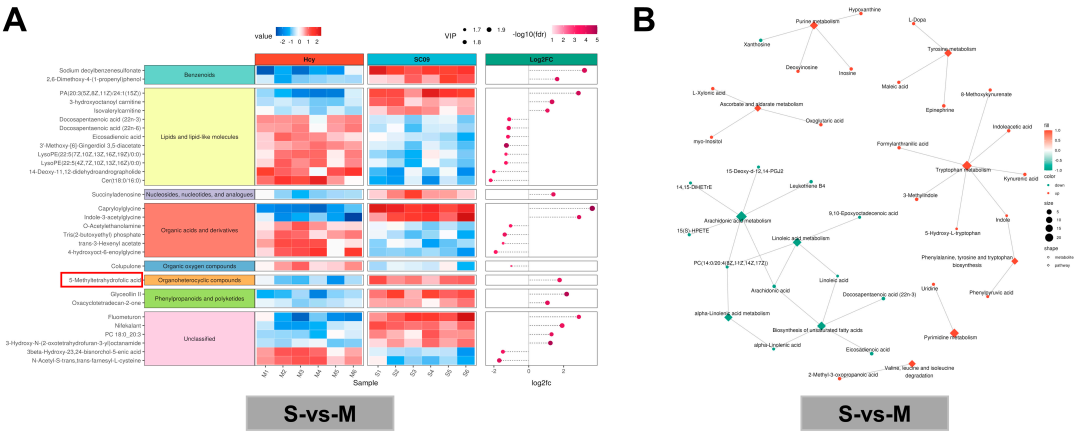

Figure S3 Metabolic Network analysis. (A) Heat map of significantly different metabolites. (B) Differential metabolite regulatory networks between Group S and Group M. ($n = 6$. Data represented as means ± SD. One-way ANOVA with Tukey's post hoc test. $p < 0.05$. VIP > 1, and abs(log2fc) > 1. The red box highlights 5-Methyltetrahydrofolic acid).

Supplemental Tables:

Table S1 Strain number information table

Serial number	Strain name	Strain number	Serial number	Strain name	Strain number
1	SC01-1	GZRD001	121	YLB06-3	GZRD121
2	SC01-2	GZRD002	122	YLB07-1	GZRD122
3	SC01-3	GZRD003	123	YLB07-2	GZRD123
4	SC01-4	GZRD004	124	YLB07-3	GZRD124
5	MR01-1	GZRD005	125	YLB07-4	*GZRD125*
6	MR01-2	GZRD006	126	PLJ05-1	GZRD126
7	SC11-1	GZRD007	127	PLJ05-2	GZRD127
8	SC11-2	GZRD008	128	PLJ05-3	GZRD128
9	SC11-3	GZRD009	129	PLJ06-1	GZRD129
10	SC12-1	GZRD010	130	PLJ06-2	GZRD130
11	SC12-2	GZRD011	131	PLJ06-3	GZRD131
12	SM01-3	GZRD012	132	XSC01-1	GZRD132
13	LJJ06-1	GZRD013	133	XSC01-2	GZRD133
14	LJJ06-2	GZRD014	134	XSC01-3	GZRD134
15	LJJ06-3	GZRD015	135	XSC02-1	GZRD135
16	YLB01-1	GZRD016	136	XSC02-2	GZRD136
17	YLB01-2	GZRD017	137	XSC02-3	GZRD137
18	YLB01-3	GZRD018	138	SC24-1	GZRD138
19	YLB02-1	GZRD019	139	SC24-2	GZRD139
20	YLB02-2	GZRD020	140	SC24-3	GZRD140
21	YLB02-3	GZRD021	141	SC25-1	GZRD141
22	YDZ01-1	GZRD022	142	SC25-2	GZRD142
23	YDZ01-2	GZRD023	143	SC25-3	GZRD143
24	YDZ01-3	GZRD024	144	SC26-1	GZRD144
25	SDJ04-1	GZRD025	145	SC26-2	GZRD145
26	SDJ04-2	GZRD026	146	SC26-3	GZRD146
27	SDJ04-3	GZRD027	147	SC27-1	GZRD147
28	SC04-1	GZRD028	148	SC27-2	GZRD148
29	SC04-2	GZRD029	149	SC27-3	GZRD149
30	SC04-3	GZRD030	150	SC28-1	GZRD150
31	SC05-1	GZRD031	151	SC28-2	GZRD151
32	SC05-2	GZRD032	152	SC28-3	GZRD152
33	SC05-3	GZRD033	153	SC29-1	GZRD153
34	SC05-4	GZRD034	154	SC29-2	GZRD154
35	SC07-3	GZRD035	155	SC29-3	GZRD155
36	SC08-1	GZRD036	156	SC30-1	GZRD156

37	SC08-2	GZRD037	157	SC30-2	GZRD157
38	SC08-3	GZRD038	158	SC30-3	GZRD158
39	SC08-4	GZRD039	159	SC31-1	GZRD159
40	SC09-1	GZRD040	160	SC31-2	GZRD160
41	SC09-1	GZRD041	161	SC31-3	GZRD161
42	SC09-1	GZRD042	162	SC32-1	GZRD162
43	SC09-1	GZRD043	163	SC32-2	GZRD163
44	SC10-1	GZRD044	164	SC32-3	GZRD164
45	SC10-2	GZRD045	165	SC33-1	GZRD165
46	SC10-3	GZRD046	166	SC33-2	GZRD166
47	SC10-4	GZRD047	167	SC33-3	GZRD167
48	LJJ03-3	GZRD048	168	XSC03-1	GZRD168
49	LJJ04-1	GZRD049	169	XSC03-2	GZRD169
50	LJJ04-2	GZRD050	170	XSC03-3	GZRD170
51	LJJ04-3	GZRD051	171	XSC04-1	GZRD171
52	LJJ04-4	GZRD052	172	XSC04-2	GZRD172
53	MR02-1	GZRD053	173	XSC04-3	GZRD173
54	MR02-2	GZRD054	174	XSC05-1	GZRD174
55	MR02-3	GZRD055	175	XSC05-2	GZRD175
56	MR02-4	GZRD056	176	XSC05-3	GZRD176
57	YLB03-4	GZRD057	177	YLB08-1	GZRD177
58	SC17-1	GZRD058	178	YLB08-2	GZRD178
59	SC17-2	GZRD059	179	YLB08-3	GZRD179
60	SC17-3	GZRD060	180	YLB09-1	GZRD180
61	SC17-4	GZRD061	181	YLB09-2	GZRD181
62	MR03-1	GZRD062	182	YLB09-3	GZRD182
63	MR03-2	GZRD063	183	YLB10-1	GZRD183
64	MR03-3	GZRD064	184	YLB10-2	GZRD184
65	MR04-1	GZRD065	185	YLB10-3	GZRD185
66	MR04-2	GZRD066	186	YLB11-1	GZRD186
67	MR04-3	GZRD067	187	YLB11-2	GZRD187
68	MR06-1	GZRD068	188	YLB11-3	GZRD188
69	MR06-2	GZRD069	189	YLB12-1	GZRD189
70	MR06-3	GZRD070	190	YLB12-2	GZRD190
71	MR09-1	GZRD071	191	YLB12-3	GZRD191
72	MR09-2	GZRD072	192	YLB13-1	GZRD192
73	MR09-3	GZRD073	193	YLB13-2	GZRD193
74	MR11-1	GZRD074	194	YLB13-3	GZRD194
75	MR11-2	GZRD075	195	XSC06-1	GZRD195
76	MR11-3	GZRD076	196	XSC06-2	GZRD196
77	MR12-1	GZRD077	197	XSC06-3	GZRD197
78	MR12-2	GZRD078	198	XSC07-1	GZRD198

79	MR12-3	GZRD079	199	XSC07-2	GZRD199
80	MR18-1	GZRD080	200	XSC07-3	GZRD200
81	MR18-3	GZRD081	201	YLB1S-1	GZRD201
82	MR20-1	GZRD082	202	YLB1S-2	GZRD202
83	MR20-2	GZRD083	203	YLB1S-3	GZRD203
84	MR20-3	GZRD084	204	YLB1S-4	GZRD204
85	MR21-1	GZRD085	205	YLB1L-1	GZRD205
86	MR21-2	GZRD086	206	YLB1L-2	GZRD206
87	MR22-1	GZRD087	207	YLB1L-3	GZRD207
88	MR22-2	GZRD088	208	YLB1L-4	GZRD208
89	MR22-3	GZRD089	209	BHG01-1	GZRD209
90	MR23-1	GZRD090	210	BHG01-2	GZRD210
91	MR23-2	GZRD091	211	BHG01-3	GZRD211
92	MR23-3	GZRD092	212	BHG01-4	GZRD212
93	MR27-1	GZRD093	213	YLB2S-1	GZRD213
94	MR27-2	GZRD094	214	YLB2S-2	GZRD214
95	MR27-3	GZRD095	215	YLB2S-3	GZRD215
96	MR29-1	GZRD096	216	YLB2S-4	GZRD216
97	MR29-2	GZRD097	217	YLB2L-1	GZRD217
98	MR29-3	GZRD098	218	YLB2L-2	GZRD218
99	MR35-1	GZRD099	219	YLB2L-3	GZRD219
100	MR35-2	GZRD100	220	YLB2L-4	GZRD220
101	MR35-3	GZRD101	221	MGC01-1	GZRD221
102	MR36-1	GZRD102	222	MGC01-2	GZRD222
103	MR36-3	GZRD103	223	MGC01-3	GZRD223
104	MR36-4	GZRD104	224	MGC01-4	GZRD224
105	MR37-3	GZRD105	225	MGC01-5	GZRD225
106	MR37-4	GZRD106	226	MGC01-6	GZRD226
107	SC18-1	GZRD107	227	DTC01-1	GZRD227
108	SC18-2	GZRD108	228	LBG01-1	GZRD228
109	SC18-3	GZRD109	229	LBG01-2	GZRD229
110	SC21-1	GZRD110	230	LBG01-3	GZRD230
111	SC21-2	GZRD111	231	LBG01-4	GZRD231
112	SC21-3	GZRD112	232	LBG01-5	GZRD232
113	SC22-1	GZRD113	233	LBG01-6	GZRD233
114	SC22-2	GZRD114	234	TR01-1	GZRD234
115	SC22-3	GZRD115	235	TR01-2	GZRD235
116	SC23-1	GZRD116	236	TR01-3	GZRD236
117	SC23-2	GZRD117	237	TR01-4	GZRD237
118	SC23-3	GZRD118	238	TR02-1	GZRD238
119	YLB06-1	GZRD119	239	TR02-2	GZRD239
120	YLB06-2	GZRD120	240	TR02-3	GZRD240

Table S2 Table of the growth status of the strain in folate-free medium

Serial number	Strain number	1st	2nd	3rd	4th	5th	6th	7th
1	GZRD001	+	-	-	-	-	-	-
2	GZRD002	+	-	-	-	-	-	-
3	GZRD003	+	-	-	-	-	-	-
4	GZRD004	+	+	+	+	+	+	+
5	GZRD005	+	+	+	+	+	+	+
6	GZRD006	+	+	+	+	+	+	+
7	GZRD007	+	+	+	+	+	+	+
8	GZRD008	+	+	+	+	+	+	+
9	GZRD009	+	+	+	+	+	+	+
10	GZRD010	+	+	+	+	+	+	+
11	GZRD011	+	+	+	+	+	+	+
12	GZRD012	+	-	-	-	-	-	-
13	GZRD013	+	-	-	-	-	-	-
14	GZRD014	+	-	-	-	-	-	-
15	GZRD015	+	-	-	-	-	-	-
16	GZRD016	+	-	-	-	-	-	-
17	GZRD017	+	-	-	-	-	-	-
18	GZRD018	+	+	+	+	+	+	+
19	GZRD019	+	+	+	+	+	+	+
20	GZRD020	+	+	+	+	+	+	+
21	GZRD021	+	+	+	+	+	+	+
22	GZRD022	+	+	+	+	+	+	+
23	GZRD023	+	-	-	-	-	-	-
24	GZRD024	+	+	-	-	-	-	-
25	GZRD025	+	+	+	+	+	+	+
26	GZRD026	+	+	+	+	+	+	+
27	GZRD027	+	+	+	+	+	+	+
28	GZRD028	+	+	+	+	+	+	+
29	GZRD029	+	+	+	+	+	+	+
30	GZRD030	+	+	+	+	+	+	+
31	GZRD031	-	-	-	-	-	-	-
32	GZRD032	+	-	-	-	-	-	-
33	GZRD033	+	-	-	-	-	-	-
34	GZRD034	+	-	-	-	-	-	-
35	GZRD035	+	+	+	+	+	+	+
36	GZRD036	+	-	-	-	-	-	-
37	GZRD037	+	+	-	-	-	-	-
38	GZRD038	+	-	-	-	-	-	-
39	GZRD039	-	-	-	-	-	-	-

40	GZRD040	-	-	-	-	-	-	-
41	GZRD041	-	-	-	-	-	-	-
42	GZRD042	+	+	+	+	+	+	+
43	GZRD043	+	+	+	+	+	+	+
44	GZRD044	+	+	+	+	+	+	+
45	GZRD045	+	+	+	+	+	+	+
46	GZRD046	+	+	+	+	+	+	+
47	GZRD047	+	+	+	+	+	+	+
48	GZRD048	+	-	-	-	-	-	-
49	GZRD049	+	-	-	-	-	-	-
50	GZRD050	+	-	-	-	-	-	-
51	GZRD051	-	-	-	-	-	-	-
52	GZRD052	-	-	-	-	-	-	-
53	GZRD053	-	-	-	-	-	-	-
54	GZRD054	+	+	+	+	+	+	+
55	GZRD055	+	-	-	-	-	-	-
56	GZRD056	-	-	-	-	-	-	-
57	GZRD057	+	-	-	-	-	-	-
58	GZRD058	-	-	-	-	-	-	-
59	GZRD059	+	-	-	-	-	-	-
60	GZRD060	+	-	-	-	-	-	-
61	GZRD061	+	-	-	-	-	-	-
62	GZRD062	-	-	-	-	-	-	-
63	GZRD063	-	-	-	-	-	-	-
64	GZRD064	-	-	-	-	-	-	-
65	GZRD065	-	-	-	-	-	-	-
66	GZRD066	+	-	-	-	-	-	-
67	GZRD067	-	-	-	-	-	-	-
68	GZRD068	-	-	-	-	-	-	-
69	GZRD069	+	+	+	+	+	+	+
70	GZRD070	-	-	-	-	-	-	-
71	GZRD071	+	-	-	-	-	-	-
72	GZRD072	+	-	-	-	-	-	-
73	GZRD073	-	-	-	-	-	-	-
74	GZRD074	-	-	-	-	-	-	-
75	GZRD075	-	-	-	-	-	-	-
76	GZRD076	-	-	-	-	-	-	-
77	GZRD077	-	-	-	-	-	-	-
78	GZRD078	+	-	-	-	-	-	-
79	GZRD079	-	-	-	-	-	-	-
80	GZRD080	-	-	-	-	-	-	-
81	GZRD081	-	-	-	-	-	-	-

82	GZRD082	-	-	-	-	-	-	-
83	GZRD083	-	-	-	-	-	-	-
84	GZRD084	-	-	-	-	-	-	-
85	GZRD085	-	-	-	-	-	-	-
86	GZRD086	-	-	-	-	-	-	-
87	GZRD087	+	-	-	-	-	-	-
88	GZRD088	-	-	-	-	-	-	-
89	GZRD089	+	+	+	+	+	+	+
90	GZRD090	+	+	+	+	+	+	+
91	GZRD091	-	-	-	-	-	-	-
92	GZRD092	-	-	-	-	-	-	-
93	GZRD093	-	-	-	-	-	-	-
94	GZRD094	-	-	-	-	-	-	-
95	GZRD095	-	-	-	-	-	-	-
96	GZRD096	+	+	+	+	+	+	+
97	GZRD097	+	+	+	+	+	+	+
98	GZRD098	+	+	+	+	+	+	+
99	GZRD099	+	+	+	+	+	+	+
100	GZRD100	+	+	+	+	+	+	+
101	GZRD101	+	+	+	+	+	+	+
102	GZRD102	+	+	+	+	+	+	+
103	GZRD103	+	+	+	+	+	+	+
104	GZRD104	+	+	+	+	+	+	+
105	GZRD105	+	+	+	+	+	+	+
106	GZRD106	+	+	+	+	+	+	+
107	GZRD107	+	+	+	+	+	+	+
108	GZRD108	+	+	+	+	+	+	+
109	GZRD109	+	+	+	+	+	+	+
110	GZRD110	+	+	+	+	+	+	+
111	GZRD111	+	+	+	+	+	+	+
112	GZRD112	+	+	+	+	+	+	+
113	GZRD113	+	+	+	+	+	+	+
114	GZRD114	+	+	+	+	+	+	+
115	GZRD115	+	+	+	+	+	+	+
116	GZRD116	+	+	+	+	+	+	+
117	GZRD117	+	+	+	+	+	+	+
118	GZRD118	+	+	+	+	+	+	+
119	GZRD119	+	+	+	+	+	+	+
120	GZRD120	+	+	+	+	+	+	+
121	GZRD121	+	+	+	+	+	+	+
122	GZRD122	+	+	-	-	-	-	-
123	GZRD123	-	-	-	-	-	-	-

124	GZRD124	-	-	-	-	-	-	-
125	GZRD125	+	+	+	+	+	+	+
126	GZRD126	+	+	+	+	+	+	+
127	GZRD127	+	+	+	+	+	+	+
128	GZRD128	+	+	+	+	+	+	+
129	GZRD129	+	+	+	+	+	+	+
130	GZRD130	+	+	+	+	+	+	+
131	GZRD131	+	+	+	+	+	+	+
132	GZRD132	+	+	+	+	+	+	+
133	GZRD133	+	+	+	+	-	-	-
134	GZRD134	-	-	-	-	-	-	-
135	GZRD135	+	+	+	+	+	+	+
136	GZRD136	+	+	+	+	+	+	+
137	GZRD137	+	+	+	+	+	+	+
138	GZRD138	+	+	+	+	+	+	+
139	GZRD139	+	+	+	+	+	+	+
140	GZRD140	+	+	+	+	+	+	+
141	GZRD141	+	+	+	+	+	+	+
142	GZRD142	+	+	+	+	+	+	+
143	GZRD143	+	+	+	+	+	+	+
144	GZRD144	+	+	+	+	+	+	+
145	GZRD145	+	+	+	+	+	+	+
146	GZRD146	+	+	+	+	+	+	+
147	GZRD147	-	-	-	-	-	-	-
148	GZRD148	-	-	-	-	-	-	-
149	GZRD149	+	+	+	+	+	+	+
150	GZRD150	+	+	+	+	+	+	+
151	GZRD151	+	+	+	+	+	+	+
152	GZRD152	+	+	+	+	+	+	+
153	GZRD153	+	+	+	+	+	+	+
154	GZRD154	+	+	+	+	+	+	+
155	GZRD155	+	+	+	+	+	+	+
156	GZRD156	+	+	+	-	-	-	-
157	GZRD157	+	+	+	+	+	+	+
158	GZRD158	+	+	+	+	-	-	-
159	GZRD159	+	+	+	+	+	+	+
160	GZRD160	+	+	+	+	+	+	+
161	GZRD161	+	+	+	+	+	+	+
162	GZRD162	+	+	+	+	+	+	+
163	GZRD163	+	+	+	+	+	+	+
164	GZRD164	+	+	+	+	+	+	+
165	GZRD165	+	+	+	+	+	+	+

166	GZRD166	+	+	+	+	+	+	+
167	GZRD167	+	+	+	+	+	+	+
168	GZRD168	+	+	+	-	-	-	-
169	GZRD169	+	+	-	-	-	-	-
170	GZRD170	-	-	-	-	-	-	-
171	GZRD171	-	-	-	-	-	-	-
172	GZRD172	-	-	-	-	-	-	-
173	GZRD173	-	-	-	-	-	-	-
174	GZRD174	-	-	-	-	-	-	-
175	GZRD175	+	+	+	+	+	+	+
176	GZRD176	+	+	+	+	+	+	+
177	GZRD177	+	+	+	+	+	+	+
178	GZRD178	+	+	+	+	+	+	+
179	GZRD179	+	+	+	-	-	-	-
180	GZRD180	-	-	-	-	-	-	-
181	GZRD181	-	-	-	-	-	-	-
182	GZRD182	+	+	+	+	+	+	+
183	GZRD183	+	+	+	+	+	+	+
184	GZRD184	+	+	+	+	+	+	+
185	GZRD185	+	+	+	+	+	+	+
186	GZRD186	+	+	+	+	+	+	+
187	GZRD187	+	+	+	+	+	+	+
188	GZRD188	+	+	+	+	+	+	+
189	GZRD189	+	+	+	+	+	+	+
190	GZRD190	+	+	+	+	+	+	+
191	GZRD191	+	+	+	-	-	-	-
192	GZRD192	+	+	+	-	-	-	-
193	GZRD193	+	+	+	+	+	+	+
194	GZRD194	+	+	+	+	+	+	+
195	GZRD195	+	+	+	+	+	+	+
196	GZRD196	+	+	+	+	+	+	+
197	GZRD197	+	+	+	+	+	+	+
198	GZRD198	+	+	+	+	+	+	+
199	GZRD199	+	+	+	+	+	+	+
200	GZRD200	+	+	+	+	+	+	+
201	GZRD201	+	+	+	+	+	+	+
202	GZRD202	+	+	+	+	+	+	+
203	GZRD203	+	+	+	+	+	+	+
204	GZRD204	+	+	+	+	+	+	+
205	GZRD205	+	+	+	+	+	+	+
206	GZRD206	+	+	+	+	+	+	+
207	GZRD207	+	+	+	+	+	+	+

208	GZRD208	+	+	+	+	+	+	+
209	GZRD209	+	+	+	+	+	+	+
210	GZRD210	+	+	+	+	+	+	+
211	GZRD211	+	+	+	+	+	+	+
212	GZRD212	+	+	+	+	+	+	+
213	GZRD213	+	+	+	+	+	+	+
214	GZRD214	+	+	+	+	+	+	+
215	GZRD215	+	+	+	+	+	+	+
216	GZRD216	+	+	+	+	+	+	+
217	GZRD217	+	+	-	-	-	-	-
218	GZRD218	-	-	-	-	-	-	-
219	GZRD219	+	+	+	+	+	+	+
220	GZRD220	+	+	+	+	+	+	+
221	GZRD221	+	+	+	+	+	+	+
222	GZRD222	+	+	+	+	+	+	+
223	GZRD223	+	+	+	+	+	+	+
224	GZRD224	+	+	+	+	+	+	+
225	GZRD225	+	+	+	+	+	+	+
226	GZRD226	+	+	+	+	+	+	+
227	GZRD227	+	+	+	+	+	+	+
228	GZRD228	+	+	+	+	+	+	+
229	GZRD229	-	-	-	-	-	-	-
230	GZRD230	-	-	-	-	-	-	-
231	GZRD231	-	-	-	-	-	-	-
232	GZRD232	+	+	+	+	+	+	+
233	GZRD233	+	+	+	+	+	+	+
234	GZRD234	+	+	+	+	+	+	+
235	GZRD235	+	+	+	+	+	+	+
236	GZRD236	+	+	+	+	+	+	+
237	GZRD237	+	+	+	+	+	+	+
238	GZRD238	+	+	+	+	+	+	+
239	GZRD239	+	+	+	+	+	+	+
240	GZRD240	+	+	+	+	+	+	+

Note: “+” indicates that it can grow.

Table S3 Growth condition tolerance table

Strain number	Comprehensive result	Strain number	Comprehensive result	Strain number	Comprehensive result
GZRD004	++ *** &&& ###	GZRD116	++ *** && ###	GZRD185	+++ *** &&& ###
GZRD005	+++ *** && ###	GZRD117	+++ *** &&& ###	GZRD186	++ ** && ##
GZRD006	++ *** &&& ###	GZRD118	++ *** && ###	GZRD187	+++ *** && ###
GZRD007	++ ** & ###	GZRD119	++ ** && #	GZRD188	+++ ** && ###
GZRD008	++ ** & ##	GZRD120	+++ *** &&& ###	GZRD189	+ * & #
GZRD009	++ ** && #	GZRD121	+++ *** &&& ###	GZRD190	+ * & #
GZRD010	++ ** & ##	GZRD125	++ ** & #	GZRD193	++ * & #
GZRD011	++ *** && ###	GZRD126	++ *** &&& ###	GZRD194	+++ ** &&& #
GZRD018	++ ** && #	GZRD127	++ ** & ##	GZRD195	+ * && #
GZRD019	++ ** & ##	GZRD128	++ *** &&& ###	GZRD196	+++ * &&& ###
GZRD020	++ ** & #	GZRD129	+++ *** &&& ##	GZRD197	+ * && #
GZRD021	++ ** & #	GZRD130	+++ *** &&& ###	GZRD198	+++ *** &&& ###
GZRD022	++ ** & #	GZRD131	+++ *** &&& ##	GZRD199	+++ *** &&& ###
GZRD025	++ ** && ###	GZRD132	+++ ** && #	GZRD200	++ * && ###
GZRD026	++ *** && ###	GZRD135	+++ *** &&& ###	GZRD201	+ * & #
GZRD027	+ ** && ##	GZRD136	+++ ** &&& ###	GZRD202	+ * & #
GZRD028	++ ** && ##	GZRD137	+++ * &&& ###	GZRD203	+ * & #
GZRD029	++ ** & ##	GZRD138	+ * & #	GZRD204	+ * & #
GZRD030	++ ** & ##	GZRD139	+ * & #	GZRD205	+++ ** &&& #

GZRD035	+++ ***<br>&& ###	GZRD140	+ *<br>& #	GZRD206	+++ **<br>&&& ###
GZRD042	++ **<br>& #	GZRD141	+ *<br>& #	GZRD207	+++ *<br>&& #
GZRD043	+++ **<br>&&& ###	GZRD142	+++ ***<br>&&& ###	GZRD208	+++ ***<br>&&& ###
GZRD044	++ **<br>&&& #	GZRD143	+ *<br>& #	GZRD209	++ *<br>&& #
GZRD045	++ **<br>&& ##	GZRD144	+ *<br>& #	GZRD210	+ *<br>&& #
GZRD046	++ **<br>& #	GZRD145	+++ ***<br>&&& ###	GZRD211	+ *<br>&& ##
GZRD047	++ ***<br>&&& ###	GZRD146	+ *<br>& #	GZRD212	+ *<br>&& ##
GZRD054	+++ ***<br>& ##	GZRD149	+++ **<br>&&& ###	GZRD213	++ **<br>&& ##
GZRD069	++ ***<br>& ###	GZRD150	++ ***<br>&& ###	GZRD214	+ *<br>&& ##
GZRD089	+++ ***<br>&& ###	GZRD151	+++ **<br>&& ###	GZRD215	+ *<br>&& ##
GZRD090	+ **<br>& ###	GZRD152	+++ ***<br>&& ###	GZRD216	+ *<br>&& ##
GZRD096	+ ***<br>&&& ##	GZRD153	+ *<br>& #	GZRD219	+++ *<br>&& ##
GZRD097	+++ ***<br>&&& ##	GZRD154	+ *<br>& #	GZRD220	+++ *<br>& ##
GZRD098	+ ***<br>&& ##	GZRD155	+ *<br>& #	GZRD221	++ *<br>&& ##
GZRD099	+ **<br>&& ##	GZRD157	+++ ***<br>&&& ##	GZRD222	++ *<br>&& ##
GZRD100	+ **<br>&& ##	GZRD159	+++ ***<br>&&& ###	GZRD223	+ *<br>&& ##
GZRD101	+ **<br>& ##	GZRD160	+ *<br>& #	GZRD224	+++ *<br>&& ##
GZRD102	+ ***<br>& ##	GZRD161	+ *<br>& #	GZRD225	+ *<br>&& ##
GZRD103	+ **<br>& #	GZRD162	+ *<br>& #	GZRD226	+++ *<br>&& ##
GZRD104	+ **<br>& #	GZRD163	+ *<br>& #	GZRD227	+++ *<br>&&& ##
GZRD105	+ **<br>& #	GZRD164	+++ ***<br>&&& ###	GZRD228	+++ **<br>&& ##

GZRD106	++ ** &&& ##	GZRD165	++ * & #	GZRD232	+++ * &&& ##
GZRD107	++ *** &&& ###	GZRD166	+ * && #	GZRD233	+++ *** &&& ###
GZRD108	++ *** && ##	GZRD167	+ * & #	GZRD234	+++ *** &&& ##
GZRD109	+ *** && ##	GZRD175	+ * & #	GZRD235	+ ** &&& ##
GZRD110	++ ** & ##	GZRD176	+ * & #	GZRD236	+++ ** &&& ##
GZRD111	++ *** && ###	GZRD177	++ *** && ##	GZRD237	+++ ** &&& ##
GZRD112	++ ** & ##	GZRD178	++ *** && #	GZRD238	+ ** &&& ###
GZRD113	++ *** &&& ###	GZRD182	+++ ** &&& #	GZRD239	+++ ** &&& ##
GZRD114	++ *** &&& ###	GZRD183	+++ ** &&& ###	GZRD240	+ *** &&& ##
GZRD115	++ *** &&& ###	GZRD184	+++ *** &&& ###		

Note: “+” indicates growth ability, “*” indicates acid tolerance, “&” indicates bile salt tolerance, “#” indicates hydrogen peroxide tolerance, and the number of symbols indicates the size of the ability.

Table S4 Strain folate production scale (μg/mL)

Strain number	Folate production	Strain number	Folate production	Strain number	Folate production
GZRD005	1.32 ± 0.05	GZRD121	1.08 ± 0.05	GZRD185	0.80 ± 0.07
GZRD006	0.81 ± 0.06	GZRD126	0.85 ± 0.10	GZRD186	0.88 ± 0.07
GZRD011	1.23 ± 0.06	GZRD129	1.02 ± 0.04	GZRD187	0.85 ± 0.07
GZRD025	0.51 ± 0.08	GZRD130	1.98 ± 0.04	GZRD188	0.94 ± 0.08
GZRD026	1.19 ± 0.02	GZRD131	1.01 ± 0.01	GZRD198	1.12 ± 0.06
GZRD028	0.28 ± 0.03	GZRD135	1.80 ± 0.04	GZRD199	1.18 ± 0.03
GZRD035	0.58 ± 0.09	GZRD136	1.07 ± 0.05	GZRD200	0.39 ± 0.08
GZRD043	2.11 ± 0.03	GZRD137	0.34 ± 0.01	GZRD206	0.16 ± 0.01
GZRD045	0.32 ± 0.07	GZRD142	1.01 ± 0.09	GZRD208	1.49 ± 0.01
GZRD047	1.08 ± 0.05	GZRD145	1.03 ± 0.04	GZRD213	0.99 ± 0.07
GZRD054	0.55 ± 0.06	GZRD149	0.71 ± 0.08	GZRD219	0.44 ± 0.01
GZRD089	0.57 ± 0.08	GZRD150	0.99 ± 0.07	GZRD224	0.48 ± 0.01
GZRD097	0.97 ± 0.01	GZRD151	0.99 ± 0.05	GZRD226	0.53 ± 0.07
GZRD111	1.02 ± 0.08	GZRD152	0.86 ± 0.01	GZRD227	1.53 ± 0.09
GZRD113	0.34 ± 0.03	GZRD157	0.93 ± 0.09	GZRD228	0.53 ± 0.06
GZRD114	0.58 ± 0.01	GZRD159	1.20 ± 0.06	GZRD233	0.55 ± 0.06
GZRD115	0.65 ± 0.04	GZRD164	0.83 ± 0.09	GZRD234	0.24 ± 0.01
GZRD117	0.64 ± 0.06	GZRD177	0.92 ± 0.03	GZRD236	0.57 ± 0.01
GZRD118	0.95 ± 0.08	GZRD183	0.82 ± 0.06	GZRD237	0.50 ± 0.07
GZRD120	1.82 ± 0.09	GZRD184	0.80 ± 0.01	GZRD239	0.34 ± 0.04

Table S5 Molecular biological identification results of 7 LAB strains

Strain number	Analysis of homology results	Homology
GZRD043	*Lactiplantibacillus plantarum* strain 3698	99.72%
GZRD120	*Enterococcus hirae* strain 13152	99.93%
GZRD130	*Lactobacillus fermentum* strain M4	99.66%
GZRD135	*Limosilactobacillus fermentum* strain CAU 237	99.66%
GZRD159	*Lactobacillus fermentum* strain 6459	96.97%
GZRD208	*Pediococcis pentosaceus* strain 2397	99.59%
GZRD227	*Pediococcis pentosaceus* strain LB	99.45%

Table S6 Folate biosynthesis-related genes

Gene ID	KO Name	KO Description
gene0740	E3.5.4.16	GTP cyclohydrolase I [EC:3.5.4.16]
gene0769	folA	dihydrofolate reductase [EC:1.5.1.3]
gene0957	queD	6-pyruvoyltetrahydropterin/6-carboxytetrahydropterin synthase [EC:4.2.3.12 4.1.2.50]
gene1955	ribBA	3,4-dihydroxy 2-butanone 4-phosphate synthase / GTP cyclohydrolase II [EC:4.1.99.12 3.5.4.25]
gene3083	ribBA	3,4-dihydroxy 2-butanone 4-phosphate synthase / GTP cyclohydrolase II [EC:4.1.99.12 3.5.4.25]
gene2350	moaB	molybdopterin adenylyltransferase [EC:2.7.7.75]
gene2351	moeA	molybdopterin molybdotransferase [EC:2.10.1.1]
gene2353	moaC	cyclic pyranopterin monophosphate synthase [EC:4.6.1.17]
gene2354	mobA	molybdenum cofactor guanylyltransferase [EC:2.7.7.77]
gene2365	moaA	GTP 3',8-cyclase [EC:4.1.99.22]
gene2367	moaE	molybdopterin synthase catalytic subunit [EC:2.8.1.12]
gene2446	folC	dihydrofolate synthase / folylpolyglutamate synthase [EC:6.3.2.12 6.3.2.17]
gene2780	folC	dihydrofolate synthase / folylpolyglutamate synthase [EC:6.3.2.12 6.3.2.17]
gene2778	folP	dihydropteroate synthase [EC:2.5.1.15]
gene2781	folE	GTP cyclohydrolase IA [EC:3.5.4.16]
gene2782	folK	2-amino-4-hydroxy-6-hydroxymethyldihydropteridine diphosphokinase [EC:2.7.6.3]
gene2783	folB	7,8-dihydroneopterin aldolase/epimerase/oxygenase [EC:4.1.2.25 5.1.99.8 1.13.11.81]

Table S7 Carbohydrate-Active enZymes

Class Definition	Genes No.
Auxiliary Activities	8
Carbohydrate Esterases	14
Glycoside Hydrolases	59
Glycosyl Transferases	33
Polysaccharide Lyases	1

Table S8 Secondary metabolite biosynthesis

Class Definition	Genes No.
NRPS	41
T3PKS	40
RiPP-like	14
cyclic-lactone-autoinducer	19
terpene	20
